# Sex-Dependent Prescription Patterns and Clinical Outcomes Associated With the Use of Two Oral Cannabis Formulations in the Multimodal Management of Chronic Pain Patients in Colombia

**DOI:** 10.3389/fpain.2022.854795

**Published:** 2022-03-24

**Authors:** Guillermo Moreno-Sanz, Alvaro Madiedo, Paula Hernandez, Janosch Kratz, Oier Aizpurua-Olaizola, Matthew R. D. Brown, Juan R. López, Jorge Patiño, Fredy O. Mendivelso

**Affiliations:** ^1^Khiron Life Sciences Spain SL, Madrid, Spain; ^2^Khiron Life Sciences SAS, Bogotá, Colombia; ^3^Khiron Europe GmbH, Frankfurt am Main, Germany; ^4^Sovereign Fields SL, San Sebastián, Spain; ^5^Zerenia Clinic, London, United Kingdom; ^6^Instituto Latinoamericano de Neurología Sistema Nervioso (ILANS)-Zerenia Clinic, Bogotá, Colombia

**Keywords:** cannabis, cannabinoids, oral extracts, chronic pain, sex, Zerenia, Khiron, Colombia

## Abstract

To date, the therapeutic use of cannabinoids in chronic pain management remains controversial owing to the limited clinical evidence found in randomized clinical trials (RCTs), the heterogeneous nature of the clinical indication, and the broad range of cannabis-based medicinal products (CBMPs) used in both experimental and observational clinical studies. Here we evaluate patient-reported clinical outcomes (PROMS) in a cohort of adult patients, diagnosed with chronic pain of diverse etiology, who received adjuvant treatment with oral, cannabis-based, magistral formulations between May and September 2021 at the Latin American Institute of Neurology and Nervous System (ILANS-Zerenia) in Bogotá, Colombia. During this period, 2,112 patients completed a PROMS questionnaire aimed at capturing the degree of clinical improvement of their primary symptom and any potential side effects. Most participants were female (76.1%) with an average age of 58.7 years old, and 92.5% (1,955 patients) reported some improvement in their primary symptom (*p* < 0.001). Two monovarietal, full-spectrum, cannabis formulations containing either cannabidiol (CBD 30 mg/mL; THC <2 mg/mL) or a balanced composition (THC 12 mg/mL; CBD 14 mg/mL) accounted for more than 99% of all prescriptions (59.5 and 39.8%, respectively). The degree of improvement was similar between both formulations, although males reported less effectiveness in the first 4 weeks of treatment. Sex-specific differences were also found in prescription patterns, with male patients increasing the intake of the balanced chemotype overtime. For many patients (71.7%) there were no adverse side effects associated to the treatment and those most reported were mild, such as somnolence (13.0%), dizziness (8.1%) and dry mouth (4.2%), which also appeared to fade over time. Our results constitute the first real-world evidence on the clinical use of medicinal cannabis in Colombia and suggest that cannabis-based oral magistral formulations represent a safe and efficacious adjuvant therapeutic option in the management of chronic pain.

## Introduction

*Cannabis sativa* L. is one of the oldest plants cultivated by humanity and its medicinal and ethnobotanical properties have been exploited for centuries by many different ancient cultures (1). The therapeutic value of cannabis extracts as analgesic and muscle relaxant was introduced to occidental medicine in the mid-19th century. At the beginning of the 20th century, cannabis-based medicinal products (CBMPs), including liquid and solid cannabis extracts, appeared in many western pharmacopeias and were broadly commercialized by laboratories around the world (including Merck, Bristol-Meyers Squibb and Eli Lilly) as an analgesic, anti-inflammatory, and anti-spastic medication (2). The medical use of CBMPs started to decline in the 1930's, partially due to their marked variability in both composition and pharmacological effects among patients, but mainly owing to the passing of the first international drug control treaties that restrained the manufacturing and trade of cannabis preparations, virtually prohibiting any further research into the clinical applications of CBMPs (3). Because of this international regulation, the Colombian government banned the cultivation of cannabis in 1939 (4). Shortly after, two main cannabinoids, the pharmacologically active molecules unique to the cannabis plant, were first identified (5, 6). The isolation of Δ9-tetrahydrocannabinol (THC) (7) and cannabidiol (CBD) (8) prompted a medical chemistry campaign that led to the discovery, in the early 1990s, of the endocannabinoid system (ECS), the pharmacological target for cannabinoids in the human body (9). The ECS is a complex physiological system composed by two G-protein coupled receptors, their lipid-derived endogenous ligands (endocannabinoids) and the enzymes responsible for the synthesis and degradation of such endocannabinoids (10). The ECS is evolutionarily well-conserved in all vertebrates and regulates many aspects of human physiological, behavioral, immunological and metabolic functions (11), including the regulation of nociception (12).

To date, THC and CBD remain the only clinically approved cannabinoids for the therapeutic modulation of the ECS. Cannabinoid-based medications have been shown to reduce chemotherapy-induced nausea and vomiting and to palliate cachexia and wasting syndrome in patients with HIV (THC/dronabinol, Marinol® and Syndros®), alleviate spasticity and neuropathic pain in patients with multiple sclerosis (THC/CBD, Sativex®), and control seizures in pediatric refractory epileptic syndromes such as Dravet, Lennox-Gastaut or tuberous sclerosis (CBD, Epidiolex®) (13). Besides these market authorized products, many jurisdictions have now permitted the medicinal use of cannabis for therapeutic purposes thus allowing for the coexistence of these medications with other presentations containing cannabinoids, such as cannabis flos (dried flowering tops) for inhalation, tinctures and capsules (14). Over the last 4 years, medicinal cannabis in the form of non-sterile oral formulations prepared on prescription for individual named patients has been available in Colombia via pharmacy compounding, under the regulations of Decree 613 of 2017. These magistral formulations must comply with all regulations applicable to pharmaceutical manufacturing and specify the content of THC and CBD as active ingredients (4).

Chronic pain is, across jurisdictions, the most common presenting complaint for which patients seek treatment with medicinal cannabis (15). However, the therapeutic use of cannabinoids in chronic pain management remains controversial owing to the limited clinical evidence found in randomized clinical trials (RCTs) using approved medications such as sativex and dronabinol, the heterogeneous etiology of chronic pain states, and the broad range of CBMPs used in both experimental and observational clinical studies (16). Because of this limited evidence base, clinical guidelines for treating chronic pain patients with medicinal cannabis rely heavily on meta-analyses of existing literature, clinical experience accrued in jurisdictions with long-standing medicinal cannabis regulations (such as Canada or Israel), and expert medical opinion. Many recent meta-analyses have reached inconsistent conclusions, with some reporting significant or robust evidence for the efficacy of medicinal cannabis to treat chronic pain (17, 18) while others demonstrate only very low-quality evidence for statistically and clinically meaningful reductions in pain intensity highlighting the significant research gaps which exist (19, 20). Improvements in pain scores observed in RCTs, although generally small and potentially accompanied by the presence of placebo effects in the comparative arm, are nevertheless thought to be related to the use of CBMPs containing THC, particularly in patients with neuropathic pain (21–24). It is therefore intriguing that several experts panels have recently supported the choice of CBD-predominant CBMPs to initiate the treatment of patients experiencing chronic pain (25, 26), an approach clearly aimed at promoting safety over efficacy considering that (i) side effects observed with medicinal cannabis are primarily attributable to THC (for example somnolence, dizziness), and (ii) the clinical evidence for the analgesic potential of CBD is minimal (27). In order to assess the translational value of these expert recommendations into the clinical setting we investigated the short-, mid-, and long-term impact of two, well-defined, oral cannabis-based formulations on patient-reported outcome measures (PROMS) from a cohort of adult patients diagnosed with chronic pain of diverse etiology. This cohort received medicinal cannabis as part of an integrative care regime at the Latin American Institute of Neurology and Nervous System (ILANS-Zerenia) in Bogotá, Colombia, between May and September of 2021.

## Materials and Methods

### Study Procedures

An observational retrospective cohort study was conducted. The medical records of patients receiving treatment with CBMPs at the ILANS-Zerenia clinic in Bogotá between May and September of 2021 were reviewed. Study procedures were approved by the Institutional Scientific Committee and Research Ethics Committee of Universidad El Bosque (Act No.012-2021). At follow-up visits, all patients were encouraged to respond to a short PROMS questionnaire which monitored the degree of clinical improvement, as well as the occurrence of any side effects. First, participants were asked to detail the primary symptom they were receiving medicinal cannabis for, which CBMPs they were currently taking, and if they had experienced any improvement of their primary symptom since commencing treatment (Yes/No answer). Participants reporting improvement were then asked to rate (0–100) their current illness score in relation to their pre-treatment baseline, with zero being “no improvement” and 100 being “total improvement of primary symptom” in a Single Assessment Numeric Evaluation (SANE) (28). Finally, all participants were asked if they had experienced any of the following side effects in relation to their treatment with CBMPs: anxiety, headache, tachycardia, somnolence, dizziness, dry mouth, diarrhea, euphoria, fatigue, blurred vision, cognitive effects, hypotension, or none (dichotomous answer Yes/No for each symptom listed). Physicians systematically registered the responses into the medical record. Responses to the SANE were clustered into four groups by increasing degree of improvement: residual (0–10), slight (20–40), moderate (50–70), and robust (80–100). PROMS were analyzed by (i) the sex of the patient; (ii) prescribed CBMPs; and (iii) duration of treatment at the time of responding the PROMS questionnaire (<4, 4–12, 13–26 weeks, and more than 26 weeks). For patients reporting more than one PROMS questionnaire during the study period, only the initial one was considered.

### Cannabis-Based Magistral Formulations

Four oral CBMPs with varying concentrations of THC and CBD were available to prescribers. Magistral formulations were prepared with monovarietal, full-spectrum extracts from legally sourced cannabis varieties registered by Khiron Life Sciences at the Colombian Institute of Agriculture (ICA). Cannabis flowering tops were extracted with supercritical CO_2_ (2,000 psi) and winterized with cold ethanol to eliminate vegetable waxes. Final products were prepared by diluting cannabis extracts to the specified concentrations using sesame oil and ethanol as excipients, sucralose, and flavoring agents. Table 1 summarizes the chemotype, cannabis cultivar used, the relative amounts of THC and CBD in the final formulation and the major terpenes found in each cultivar. For additional information, certificates of analysis of both final CBMPs and original cannabis cultivars are provided as Supplementary Material.

**Table 1:** Chemical specifications of magistral formulations available to prescribers at ILANS-Zerenia clinic.

**# CBPMs**	**Chemotype**	**Cannabis variety**	**(THC) mg/mL**	**(CBD) mg/mL**	**Major terpenes (in flos)**
FM-001	I	TA-3-008	20	<1	β-caryophyllene, α-humulene
FM-002	II	WW-3-011	12	14	β-myrcene, pinene
FM-003	III	FT-1-009	<2	30	β-myrcene, limonene
FM-004	III	FT-1-009	<2	100	β-myrcene, limonene

### Statistical Analysis

Descriptive statistics were performed for demographic data, clinical outcomes measures and prescription patterns. Results are expressed as mean and/or mean ± standard deviation (SD). Log-linear regressions were used to compare prescription patterns and occurrence of side effects between group variables (sex and chemotype). ANOVAs were used to compare patient-reported scores of medical improvements between group variables (sex, chemotype, and duration of treatment). Clinical data was obtained from the hospital management system Gomedisys (Bogotá, Colombia) and analyzed using the Jamovi free software V2.2.2.

## Results

### Participant Demographics

**Figure 1:**
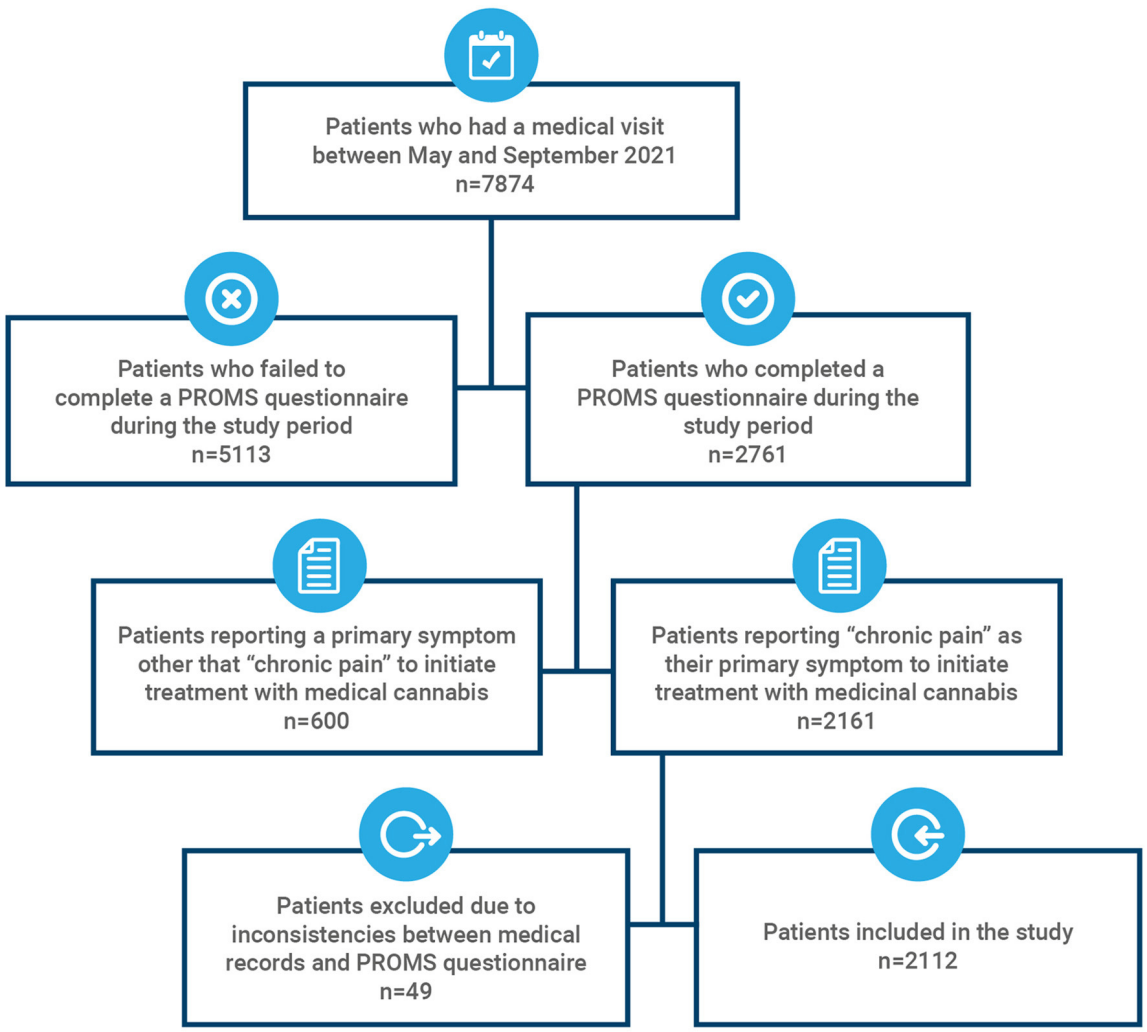
Data inclusion flowchart.

A total of 7,874 patients receiving medicinal cannabis as part of their treatment regime had a follow- up consultation during the study period, and 2,761 completed the PROMS questionnaire during one of their follow-up appointments. Of those, 2,161 (78.3%) reported “chronic pain” as their primary symptom to receive treatment with medicinal cannabis. Forty-nine patients presenting inconsistencies between their PROMS questionnaire and their medical or pharmacy records (for example, the patient had not commenced the treatment at the time of completing the questionnaire or reported a primary symptom which did not match the diagnosis) were excluded from the study (Figure 1). Demographics of the cohort are summarized in Table 2. A total of 2,112 patients were included in the study, a majority of which were female (76.1%) with an average age of 58.7 years old, ranging from 18 to 98. Older adults (>65) represented 32.29% of participants. This cohort closely represents the entire clinical population of 7,874 patients, which was composed primarily of females (73.04%) with an average age of 59.8 ± 15.4 and 38.5% of the population being older adults. Most patients accessing the pain department of the clinic were affiliated to the Colombian general healthcare system through a regime either contributive (78.2%), subsidized (2.8%) or occupational (0.1%), and were referred by their main healthcare provider to receive a specialized treatment with medicinal cannabis. Additionally, self-referrals represented 18.9% of patients. Participants who reported experiencing “chronic pain” as their primary indication were diagnosed mainly in three categories according to the WHO international statistical classification of diseases and related health problems (ICD-10): (i) unspecified pain, (ii) diseases of the musculoskeletal system and connective tissue, and (iii) nervous system diseases.

**Table 2:** Demographic characterization of the study cohort.

**Variable**	**Female**	**Male**	**Total**
	***n* = 1,608**	***n* = 504**	***n* = 2,112**
	**76.14%**	**23.86%**	
Age, mean (±SD)	59.0 (±13.9)	57.9 (±14.4)	58.7 (±14.1)
Percentage of older adults (>65)	32.59%	31.35%	32.29%
**Type of insurance**, ***n*** **(%)**			
Social security (contributive)	1,259 (78.3)	392 (77.8)	1651 (78.2)
Subsidized by the State	45 (2.8)	15 (3.0)	60 (2.8)
Particular	303 (18.8)	96 (19.0)	399 (18.9)
Laboral risk insurance	1 (0.1)	1 (0.2)	2 (0.1)
**Diagnosis ICD-10**, ***n*** **(%)**			
Unspecified pain	941 (58.5)	360 (71.4)	1,301 (61.6)
Diseases of the
musculoskeletal system and
connective tissue	471 (29.3)	89 (17.7)	560 (26.5)
Nervous system diseases	145 (9.0)	40 (7.9)	185 (8.8)
Other	51 (3.2)	15 (3.0)	66 (3.1)

### Prescription Patterns

**Figure 2:**
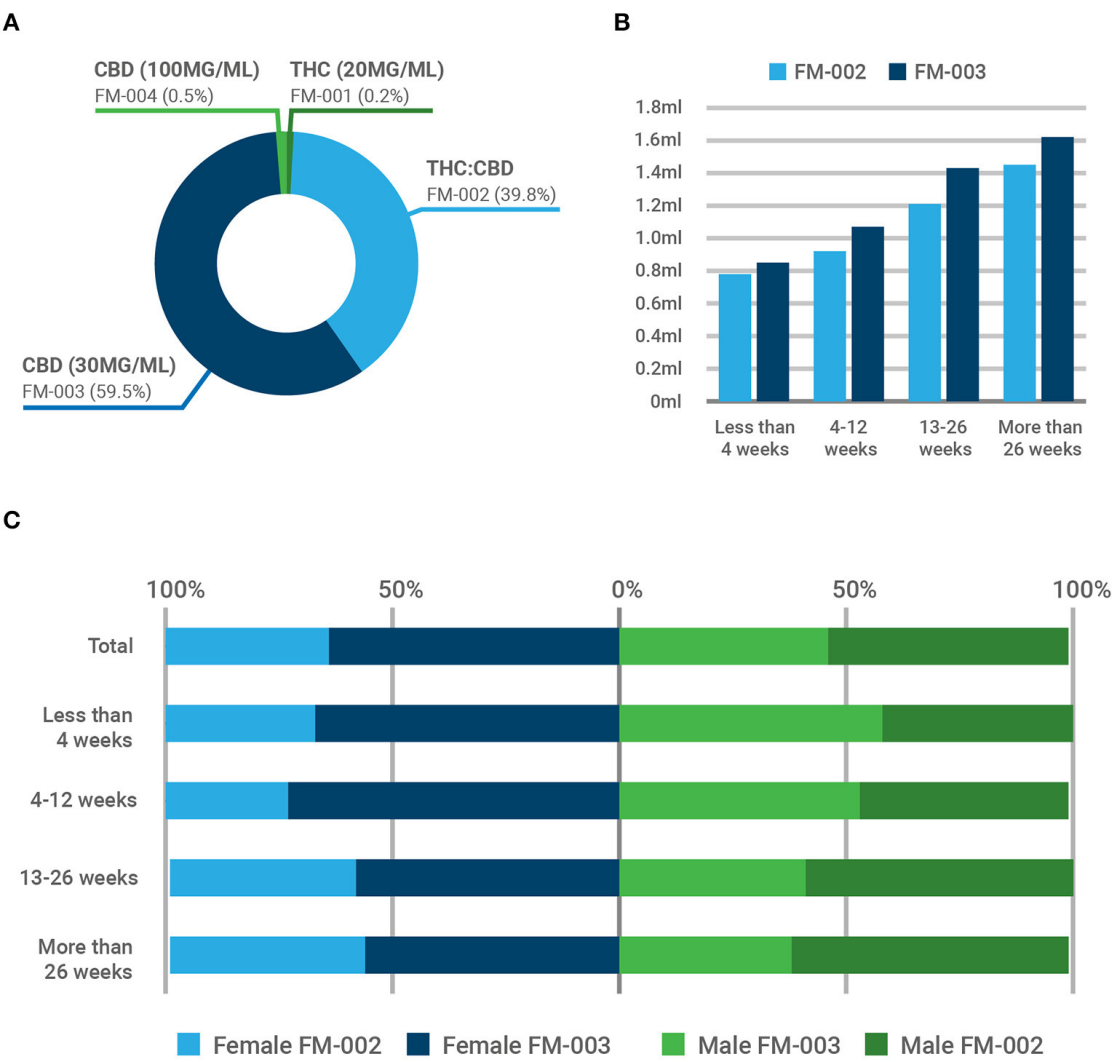
Prescription patterns of cannabis-based magistral formulations at ILANS-Zerenia clinic. Four oral CBMPs with varying concentrations of THC and CBD were available to prescribers. **(A)** Percentage of participants prescribed with each magistral formulation. **(B)** Mean daily intake (in mL) of FM-002 (light blue) and FM-003 (dark blue). **(C)** Temporal evolution of the prescription patterns between females (blue bars) and males (green bars). Overall, FM-002 was preferentially prescribed to men and FM-003 to woman (*p* < 0.001).

Out of the four magistral formulations available to prescribers, the chemotype-I formulation containing only THC (FM-001: THC = 2 mg/mL) and the epidiolex-like formulation (FM-004: CBD = 100 mg/mL) where only marginally used (Figure 2A). On the contrary, the chemotype-III formulation containing primarily cannabidiol (FM-003: CBD = 30 mg/mL; THC <2 mg/mL) and the chemotype-II or balanced composition (FM-002: THC = 12 mg/mL; CBD = 14 mg/mL) accounted for more than 99% of all prescriptions (59.5 and 39.8%, respectively). Initiating a treatment with CBMPs, especially if they contain THC, usually involves a titration phase in which the dosing is slowly and steadily increased every few days until therapeutic goals are achieved (for example, symptom control). This approach aims to minimize the occurrence of side effects. Figure 2B depicts the average volume of each CBMP (in mL) consumed by patients depending on the duration of treatment. The average dose of THC consumed by patients treated with FM-002 ranged between 9.4 and 17.4 mg per day, and the average dose of CBD on patients treated with FM-003 ranged from 25.6 to 48.5 mg per day. Interestingly, we found significant sex bias (*Z* = 6.807, *p* < 0.001) in the prescription patterns of these two CBMPs, as depicted in Figure 2C. Overall, 64% of females, but only 46% of male patients, were prescribed with FM-003 (for absolute values see Table 3). This relative distribution evolved over time, depending on the duration of treatment. For females, treatment with medicinal cannabis was initiated mainly with CBD (67%), reaching a maximum prescription in those patients receiving treatment for a duration of between 4 and 12 weeks (72%). This proportion was reduced in patients enrolled in the program for longer periods yet remained the preferred therapeutic option for females (58 and 56% in patients treated for 13–26 weeks or longer, respectively). Conversely, although FM-003 represented the starting CBMP of choice for 58% of newly prescribed male patients, the proportion compared to FM-002 diminished over time. In fact, those male patients enrolled in the medicinal cannabis program for longer than 12 weeks were treated preferentially with the THC-containing formulation (FM-002), which was primarily prescribed to male patients receiving medicinal cannabis treatment for 13–26 weeks or longer (59 and 61%, respectively). This sexual divergence appears to correlate with a lower perceived efficacy of the CBD product by male patients compared to the formulation containing both THC and CBD.

**Table 3:** Occurrence of adverse side effects for each magistral formulation.

	**Magistral formulation**				**Total** ***N* = 2,112**
	**FM-001**	**FM-002**	**FM-003**	**FM-004**	
	***n* = 4**	***n* = 856**	***n* = 1,241**	***n* = 11**	
**Gender**, ***n*** **(%)**					
Female	3 (75)	586 (68.4)	1,011 (81.4)	8 (72.7)	1.608 (76.1)
Male	1 (25)	270 (31.5)	230 (18.5)	3 (27.2)	504 (23.8)
**Adverse side effects**, ***n*** **(%)**					
None	3 (75)	547 (63.9)	954 (76.8)	11 (100)	1,515 (71.7)
Somnolence	1 (25)	145 (16.9)	129 (10.3)	0 (0)	275 (13.0)
Dizziness	0 (0)	104 (12.1)	67 (5.39)	0 (0)	171 (8.09)
Dry mouth	0 (0)	35 (4.08)	55 (4.43)	0 (0)	90 (4.26)
Headache	0 (0)	7 (0.81)	10 (0.80)	0 (0)	17 (0.80)
Anxiety	0 (0)	5 (0.58)	9 (0.72)	0 (0)	14 (0.66)
Tachycardia	0 (0)	2 (0.23)	6 (0.48)	0 (0)	8 (0.37)
Diarrhea	0 (0)	3 (0.35)	3 (0.24)	0 (0)	6 (0.28)
Mild headache	0 (0)	2 (0.23)	4 (0.32)	0 (0)	6 (0.28)
Cognitive side effects	0 (0)	2 (0.23)	1 (0.08)	0 (0)	3 (0.14)
Euphoria	0 (0)	2 (0.23)	1 (0.08)	0 (0)	3 (0.14)
Fatigue	0 (0)	2 (0.23)	0 (0)	0 (0)	2 (0.09)
Hypotension	0 (0)	0 (0)	2 (0.16)	0 (0)	2 (0.09)
Blurred vision	0 (0)	0 (0)	0 (0)	0 (0)	0 (0)

### Outcome Measures

#### Improvement of Primary Symptom

**Figure 3:**
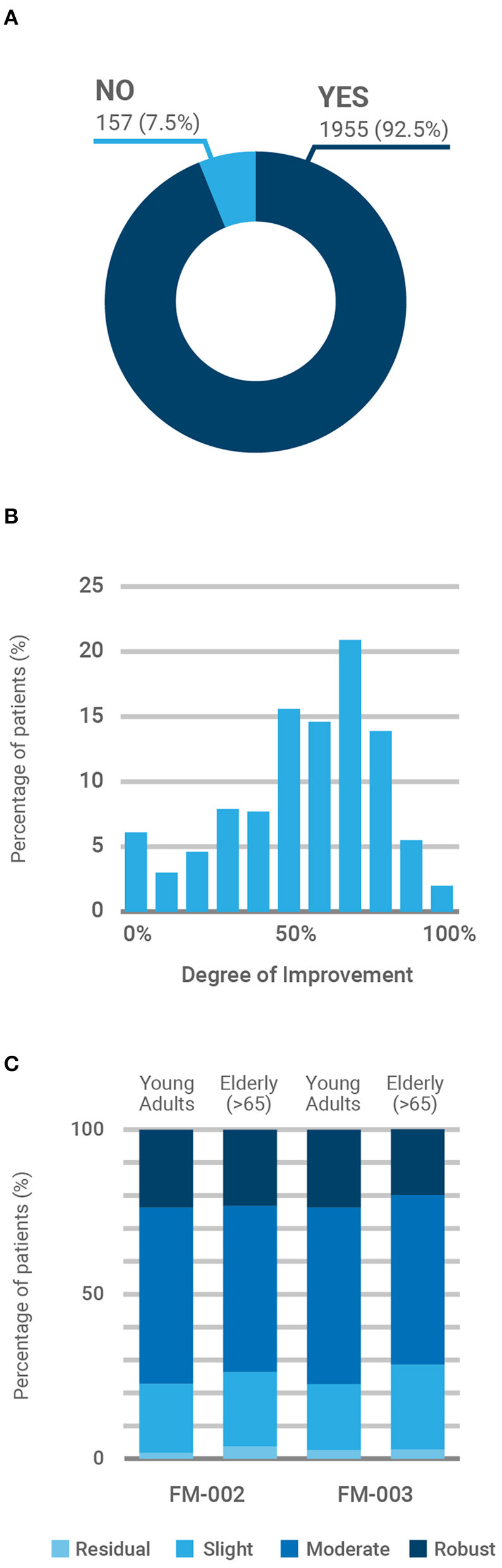
Patient-reported improvement of primary symptom (chronic pain). **(A)** Percentage of patients reporting any degree of improvement (yes/no) in their primary symptom (*p* < 0.001). **(B)** Participants' responses (*N* = 2,112) to the 11-point Single Assessment Numeric Evaluation (SANE), rating their current illness score in relation to their pre-treatment baseline, with zero being “no improvement” and 100 being “total improvement of primary symptom”. **(C)** Reported degree of improvement by age group (young adult <65 vs. elderly >65) of patients prescribed with FM-002 (*n* = 856) and FM-003 (*n* = 1,241). To facilitate visual interpretation, responses to the SANE were clustered into four groups by increasing degree of improvement: residual (0–10), slight (20–40), moderate (50–70), and robust (80–100). Participants prescribed with FM-001 and FM-004 were marginal (<1%) and responses were not included in the analysis to avoid confusion.

**Figure 4:**
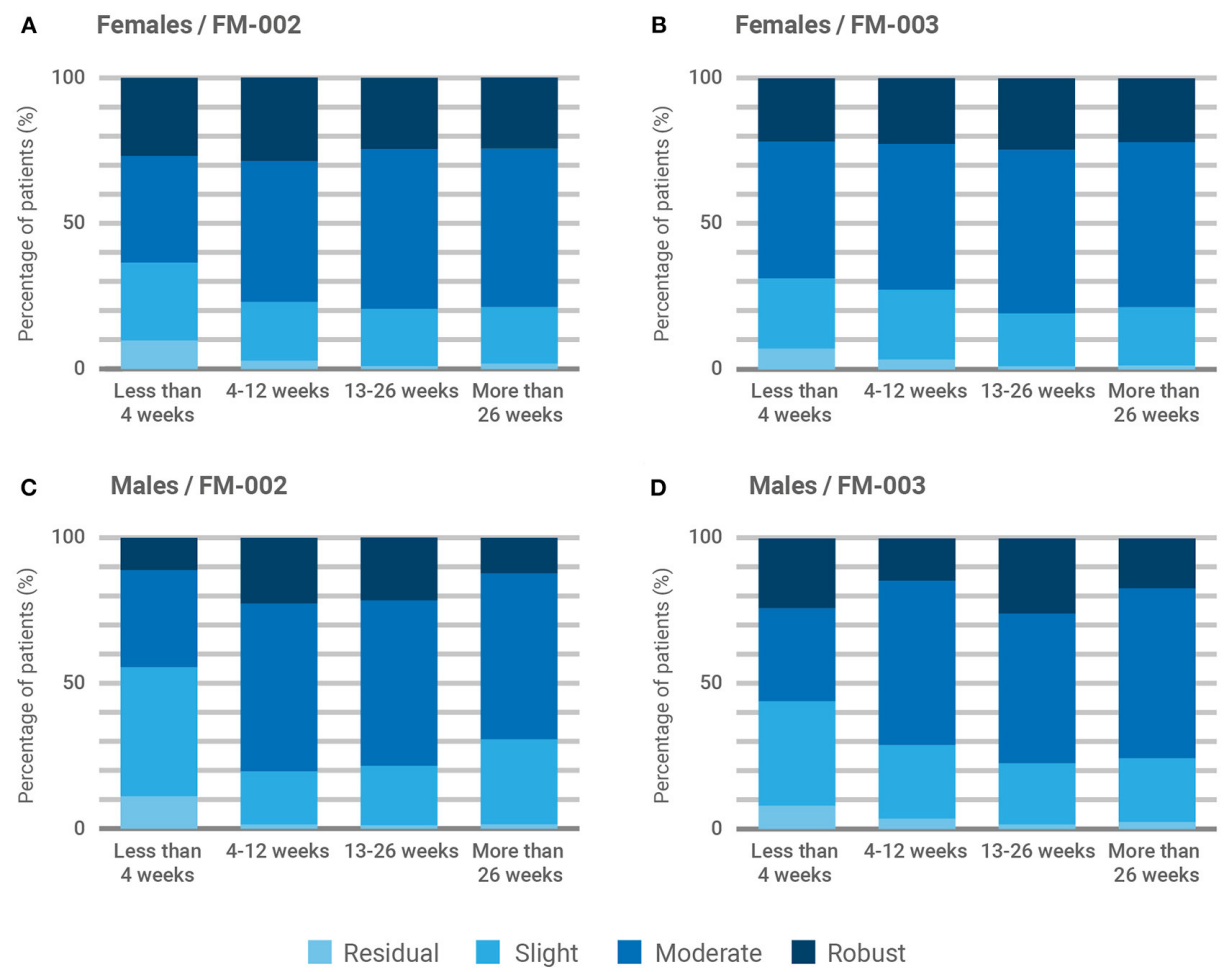
Influence of sex and treatment duration over patient-reported improvement of primary symptom. Responses from participants were clustered into four groups by increasing degree of improvement, residual (0–10), slight (20–40), moderate (50–70), and robust (80–100), and are presented by sex and chemotype: **(A)** Female participants prescribed with FM-002 (*n* = 586); **(B)** Female participants prescribed with FM-003 (*n* = 1,011); **(C)** Male participants prescribed with FM-002 (*n* = 270); **(D)** Male participants prescribed with FM-003 (*n* = 230).

Most participants (92.7%, Figure 3A) reported some degree of clinical improvement in their chronic pain following medicinal cannabis treatment. The average improvement reported was 54.7 ± 24.8% (*p* < 0.001) and patient responses to the SANE presented a gaussian distribution centered on the response indicating clinical improvement of 70% (Figure 3B). To facilitate visualization and interpretation of results, the degree of improvement was clustered in four categories: residual (0–10), slight (20–40), moderate (50–70), and robust (80–100). Therefore, the last two categories represent a reported medical improvement >50%. Because FM-001 and FM-004 were rarely prescribed, we focused our comparative analysis in the results reported for FM-002 and FM-003. As shown in Figure 3C, the overall reported degree of medical improvement was similar between both CBMPs, with more than 75% of patients reporting either a moderate or robust reduction of chronic pain. Significant differences in the degree of improvement reported by age groups (young adults vs. elderly) were not found (χ^2^ = 0.335; *P* = 0.563), although patients older than 65 appeared to report slightly less benefit from the treatment (Figure 3C). The contribution of the sex of the patient and the duration of treatment on these outcomes was also examined (Figure 4). ANOVA analysis failed to reveal statistically significant differences between chemotypes (χ^2^ = 0.0756; *P* = 0.783), patient sex (χ^2^ = 3.75; *P* = 0.053) or duration of treatment (χ^2^ = 3.06; *P* = 0.383). However, female patients reported a steady improvement in pain control after commencing treatment (Figures 4A,B) while males reported less benefit from medicinal cannabis treatment in the first 4 weeks (Figures 4C,D). Further, FM-002 was reported as slightly less effective than FM-003 on the first month of treatment for both males and females (Figures 4A,C) although this trend disappeared after the initial 4 weeks. Of note, females reported the most pronounced improvement of chronic pain (29%) with FM-002 between 4 and 12 weeks of treatment (Figure 4A).

#### Occurrence of Adverse Side Effects

**Figure 5:**
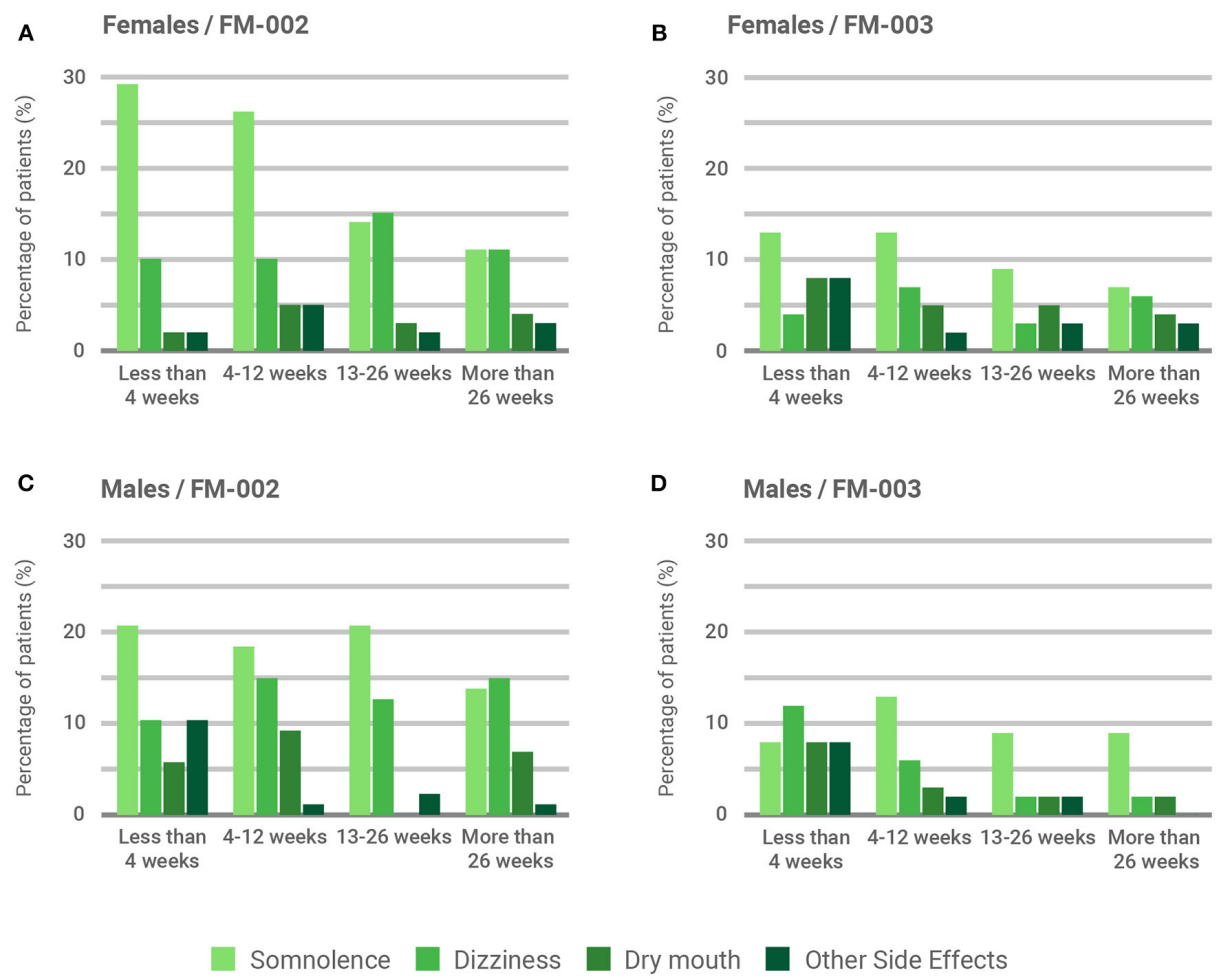
Influence of sex and treatment duration over patient-reported adverse side effects. Percentage of occurrence of the three most-frequently reported adverse side effects (somnolence, dizziness, and dry mouth) is illustrated. Remaining adverse side effects (see Table 3) are clustered as “other”. Results are presented by sex and chemotype: **(A)** Female participants prescribed with FM-002 (*n* = 586); **(B)** Female participants prescribed with FM-003 (*n* = 1,011); **(C)** Male participants prescribed with FM-002 (*n* = 270); **(D)** Male participants prescribed with FM-003 (*n* = 230).

The majority of participants (71.7%) did not report significant adverse side effects associated with treatment with CBMPs. Those side effects reported were mild, such as somnolence (13%), dizziness (8.1%) and dry mouth (4.3%). Of note, serious adverse events requiring hospitalization or medical intervention were not reported. Table 3 summarizes all side effects reported by participants, indicating a higher prevalence of neurological side effects (somnolence and dizziness) associated with the THC-containing FM-002, compared to FM-003 (*p* < 0.05). This feature becomes even more apparent when results are filtered by sex and duration of treatment (Figure 5), with somnolence being the most common side effect associated to FM-002 in both males and females (Figures 5A,C). However, although females reported this side effect significantly more frequently than males in the first 4 and 12 weeks of treatment (29 and 26% vs. 18 and 16%, respectively, *p* < 0.05), its incidence diminished as the duration of treatment increased, eventually becoming equal between sexes. In contrast, dizziness presented a lower incidence from the outset, however patients continued to report similar levels regardless of the duration of treatment. Additionally, FM-003 was associated with lower reporting of side effects, mainly somnolence, which followed a similar diminishing trend in females over time while remaining unrelated to the duration of treatment in male patients (Figures 5B,D). Dry mouth was equally reported for both CBMPs by males and females. Other side effects (Table 3) were mainly reported by patients within the first 4 weeks of treatment (Figures 5A,C,D) and tended to reduce in intensity over the course of treatment.

## Discussion

The therapeutic use of cannabinoids in chronic pain management remains controversial mainly due to the legal barriers that historically prevented clinical researchers from accessing cannabis-based products for medicinal use. This study represents the first published description of the use of cannabis-based magistral preparations in Colombia since the country instated the regulatory framework granting eligible patients safe and informed access to medicinal cannabis and its derivatives. Participants were patients of the ILANS-Zerenia clinic, the first integrative healthcare provider based in Bogotá with a specialized team of physicians offering cannabis-based medicinal products as an adjuvant therapy to eligible patients, in combination with physiotherapy and pain-related psychotherapy. Magistral formulations used in this study were provided by Khiron Life Sciences, a licensed manufacturer of cannabis derivatives authorized by the Colombian Health Ministry. This clinical population was primarily composed of females of relatively advanced age and the most common indication for which patients received medicinal cannabis was chronic pain. Unspecified pain was more common in males whilst musculoskeletal pain was more frequent in females.

Analysis of prescription patterns revealed that two CBMPs were almost exclusively used by prescribing physicians: a chemotype-III formulation containing predominantly CBD (FM-003) and a chemotype-II formulation with a balanced composition of THC and CBD (FM-002). The chemotype-I formulation (FM-001), a full-spectrum extract containing 20 mg/mL of THC, was the least used among doctors. This observation is intriguing considering that the available medical evidence on cannabinoid therapeutics supports the efficacy of THC-containing products for the treatment of pain (23, 24). However, since the approval in Canada of Sativex®, an oromucosal spray that contains 27 mg of THC and 25 mg of CBD per mL, for the management of neuropathic pain associated with multiple sclerosis (MS), the notion of dronabinol (isolated THC) being too aversive has favored the utilization of balanced formulations that include equimolar amounts of CBD (14). This approach has been proposed to reduce unwanted neurological and psychological side effects associated with unabated THC (29). Further, Sativex® demonstrated superior efficacy compared to both THC-predominant extracts and placebo at reducing pain in RCTs thus suggesting that the CBD component contributes an analgesic effect (21). Nevertheless, our results showed that most participants, especially females, initiated their treatment with a CBD-predominant product containing <2 mg/mL of THC. This is in strong agreement with (i) a recent observational study including 9,766 Canadian older adults receiving treatment with medicinal cannabis in which 83.6% were receiving CBMPs containing only or mostly CBD (30); and (ii) the recommendations by an industry-sponsored multidisciplinary group of global experts who recently developed a set of clinical guidelines for the dosing and administration of cannabinoids to treat chronic pain through a modified Delphi process (26).

Despite public perception and preclinical promise, clinical evidence supporting the efficacy of CBD as an analgesic is virtually non-existent (31, 32). However, we found that the improvement in chronic pain reported by patients was identical for both chemotypes, an unexpected observation that could be due to the limitations of our study. By using a convenience sample, our results may over emphasize beneficial responses due to the preferential withdrawal of patients with poor medical outcomes (33). Indeed, the proportion of patients prescribed the CBD-predominant formulation diminished over time which, although partially due to participants switching chemotypes from FM-003 to FM-002, was primarily related to patients prescribed with FM-003 abandoning the treatment. This was observed more frequently in male patients. Human laboratory experiments suggest that patient's expectations play a major role in both the analgesic and anxiolytic effects of CBD (34, 35). Therefore, overemphasizing the analgesic potential of CBD could result into negative treatment outcomes when such expectations are not met (36). Additionally, CBD has been proposed to alleviate concomitant anxiety in chronic pain patients (14) which may contribute to both the self-reported benefit in primary symptoms by female participants treated with FM-003 as well as the observed sex-bias in chemotype selection, considering that females present consistently higher prevalence rates of anxiety disorders compared to males (37). In support of this explanation, Gruber and colleagues have recently reported results from a small cohort of chronic pain patients showing that the treatment with medicinal cannabis improved not only pain scores but also several measures of sleep, mood, anxiety, and quality of life. Interestingly, their results suggest that, in general, THC intake was related to pain-related improvement while CBD formulations were related to improved mood, and that reported improvements were not related to patient expectancies (38). Finally, CBD-predominant, full-spectrum cannabis extracts typically contain small amounts of THC which may contribute to their overall pharmacological effect (39). Although this is a frequent concern in unregulated markets, Colombian regulations mandate a strict compliance with product specifications. In the case of FM-003, the maximum concentration of THC allowed is 2 mg/mL, which renders a theoretical THC/CBD ratio of 1:15, although THC concentration is typically lower (Supplementary Material). Therefore, we may speculate that magistral formulations covering intermediate ratios of THC/CBD within the theoretical range defined by FM-002 (1:1) and FM-003 (1:15) could potentially improve the therapeutic index of these products (40). Alternatively, non-concomitant administration of CBD and THC has been shown to promote synergistic effects through pharmacokinetic and pharmacodynamic interactions (40), suggesting that combinations of FM-001 and FM-003 at different time intervals (e.g., night time/day time) could also represent a successful strategy to potentiate the benefits of the treatment.

Regardless of the lack of significant differences in treatment effectiveness observed between both chemotypes and the mentioned limitation of the convenience cohort, we were able to draw some relevant observations from our study. First, both males and females reported more significant benefits after the initial 4 weeks once an optimal dosing regimen was reached. This period was also associated with the highest incidence of adverse side effects. It is therefore extremely important for both patients and prescribing physicians to persist with therapy through this initial dosing adjustment phase. Establishing treatment goals during the initial consultation can be helpful to provide a structure to this process (26). Second, most observational and experimental studies investigating the efficacy of CBMPs for pain management are of relatively short duration, generally under 4 weeks (33). Our results indicate that the perceived effectiveness reported by patients was maintained on the short- (4–12 weeks), intermediate- (12–26 weeks), and long-term (>26 weeks), which is well-aligned with results from RCTs using Sativex® in which MS patients showed sustained improvements in pain and other symptoms for more than 12 months without developing tolerance (41). Finally, treatment with medicinal cannabis was generally well-tolerated, with no serious adverse events occurring. As expected, FM-002 was accompanied by a significantly higher prevalence of CNS-related side effects, such as somnolence and dizziness, which were more prominent in the first weeks of treatment (14). Of note, somnolence was predominantly reported by females compared to males during the first 12 weeks of treatment. Similar sex differences in adverse effects related to medicinal cannabis have recently been disclosed by other authors in Israel (42).

## Conclusions

Medicinal cannabis in the form of oral magistral formulations may represent a valuable option for physicians as an adjuvant therapeutic intervention in the management of chronic pain and associated comorbidities. Considering the current lack of medical guidelines, evidence accrued through real-world clinical experience can help inform best medical practices in terms of chemotype selection and dosing regime to maximize therapeutical effectiveness and tolerability. To our knowledge, this study represents the first clinical investigation of medicinal cannabis usage for chronic pain in a cohort of Colombian patients. However, given the high incidence of the pain phenotypes described in our study, the findings are generalizable across patient populations. This view is supported by the fact that our results are well-aligned with those reported from similar clinical populations by research groups working in international jurisdictions with long-standing medicinal cannabis access programs such as Israel or Canada.

## Data Availability Statement

The raw data supporting the conclusions of this article will be made available by the authors, without undue reservation.

## Ethics Statement

The studies involving human participants were reviewed and approved by Institutional Scientific Committee and Research Ethics Committee of Universidad El Bosque. Written informed consent for participation was not required for this study in accordance with the national legislation and the institutional requirements.

## Author Contributions

GM-S, FM, and PH conceptualized the study and designed the experimental protocol. JL and JP were responsible for generating the clinical data. Data was analyzed and processed by AM, GM-S, and FM. OA-O provided the analysis of cannabis inflorescences. GM-S wrote the manuscript with aid from PH, AM, MB, and JK. All authors agreed to be accountable for the content of the work.

## Funding

Research was funded by ILANS-Zerenia Clinic and Khiron Life Sciences Corp.

## Conflict of Interest

OA-O was employed by Sovereign Fields SL. This study received funding from ILANS-Zerenia Clinic and Khiron Life Sciences Corp. The funder had the following involvement with the study: GM-S, AM, PH, and JK are employees of Khiron Life Sciences, an authorized cannabis manufacturer which provided the cannabis-based magistral formulations used in this study. FM is an independent research consultant hired by ILANS-Zerenia Clinic. No other personnel or management from Khiron Life Sciences Corp. was involved in the study design, collection, analysis, interpretation of data, the writing of this article or the decision to submit it for publication. The remaining authors declare that the research was conducted in the absence of any commercial or financial relationships that could be construed as a potential conflict of interest.

## Publisher's Note

All claims expressed in this article are solely those of the authors and do not necessarily represent those of their affiliated organizations, or those of the publisher, the editors and the reviewers. Any product that may be evaluated in this article, or claim that may be made by its manufacturer, is not guaranteed or endorsed by the publisher.

## References

[1] Russo EB. History of cannabis and its preparations in saga, science, and sobriquet. Chem Biodiversity. (2007) 4:1614–48. 10.1002/cbdv.200790144 17712811

[2] Pisanti S Bifulco M. Modern history of medical cannabis: from widespread use to prohibitionism and back. Trends Pharmacol Sci. (2017) 38:195–8. 10.1016/j.tips.2016.12.002 28095988

[3] Baron EP. Comprehensive review of medicinal marijuana, cannabinoids, and therapeutic implications in medicine and headache: what a long, strange trip it's been. Headache. (2015) 55:885–916. 10.1111/head.12570 26015168

[4] Arias S Leon M Jaimes D Bustos R-H. Clinical evidence of magistral preparations based on medicinal cannabis. Pharmaceuticals. (2021) 14:1–13. 10.3390/ph14020078 33494156 PMC7909828

[5] Adams R Hunt M. Structure of cannabidiol, a product isolated from the marihuana extract of Minnesota Wild Hemp. I. J Am Chem Soc. (1940) 62:196–200. 10.1021/ja01858a058

[6] Adams R Pease DC Cain CK Baker BR Clark JH Wolff H. Conversion of cannabidiol to a product with marihuana activity. A type reaction for synthesis of analogous substances. Conversion of cannabidiol to cannabinol. J Am Chem Soc. (1940) 62:2245–6. 10.1021/ja01865a508

[7] Gaoni Y Mechoulam R. Isolation, structure, and partial synthesis of an active constituent of Hashish. J Am Chem Soc. (1964) 86:1646–7. 10.1021/ja01062a046

[8] Mechoulam R Shvo Y. Hashish. I. The structure of cannabidiol. Tetrahedron. (1963) 19:2073–8. 10.1016/0040-4020(63)85022-X 5879214

[9] Herkenham M Lynn AB Little MD Johnson MR Melvin LS De Costa BR. Cannabinoid receptor localization in brain. Proc Natl Acad Sci USA. (1990) 87:1932–36. 10.1073/pnas.87.5.1932 2308954 PMC53598

[10] Cristino L Bisogno T Di Marzo V. Cannabinoids and the expanded endocannabinoid system in neurological disorders. Nat Rev Neurol. (2020) 16:9–29. 10.1038/s41582-019-0284-z 31831863

[11] Joshi N Onaivi ES. Endocannabinoid system components: overview and tissue distribution. Adv Exp Med Biol. (2019) 1162:1–12. 10.1007/978-3-030-21737-2_1 31332731

[12] Woodhams SG Chapman V Finn DP Hohmann AG Neugebauer V. The cannabinoid system and pain. Neuropharmacology. (2017) 124:105–20. 10.1016/j.neuropharm.2017.06.015 28625720 PMC5785108

[13] Amin MR Ali DW. Pharmacology of medical cannabis. Adv Exp Med Biol. (2019) 1162:151–65. 10.1007/978-3-030-21737-2_8 31332738

[14] MacCallum CA Russo EB. Practical considerations in medical cannabis administration and dosing. Eur J Int Med. (2018) 49:12–9. 10.1016/j.ejim.2018.01.004 29307505

[15] Boehnke KF Gangopadhyay S Clauw DJ Haffajee RL. Qualifying conditions of medical cannabis license holders in The United States. Health Aff (Millwood). (2019) 38:295–302. 10.1377/hlthaff.2018.05266 30715980 PMC6398594

[16] Köstenberger M Nahler G Jones TM Neuwersch S Likar R. The role of cannabis, cannabidiol and other cannabinoids in chronic pain. The perspective of physicians. J Neuroimmune Pharmacol. (2021) 31. 10.1007/s11481-021-10010-x. [Epub ahead of print]. 34467511

[17] Whiting PF Wolff RF Deshpande S Di Nisio M Duffy S Hernandez AV. Cannabinoids for medical use: a systematic review and meta-analysis. J Am Med Assoc. (2015) 313:2456–73. 10.1001/jama.2015.6358 26103030

[18] Sainsbury B Bloxham J Pour MH Padilla M Enciso R. Efficacy of cannabis-based medications compared to placebo for the treatment of chronic neuropathic pain: a systematic review with meta-analysis. J Dental Anesth Pain Med. (2021) 21:479. 10.17245/jdapm.2021.21.6.479 34909469 PMC8637910

[19] Mücke M Phillips T Radbruch L Petzke F Häuser W. Cannabis-based medicines for chronic neuropathic pain in adults. Cochr Database Syst Rev. (2018) 3:CD012182. 10.1002/14651858.CD012182.pub2 29513392 PMC6494210

[20] Stockings E Campbell G Hall WD Nielsen S Zagic D Rahman R. Cannabis and cannabinoids for the treatment of people with chronic noncancer pain conditions: a systematic review and meta-analysis of controlled and observational studies. Pain. (2018) 159:1932–54. 10.1097/j.pain.0000000000001293 29847469

[21] Johnson JR Burnell-Nugent M Lossignol D Ganae-Motan ED Potts R Fallon MT. Multicenter, double-blind, randomized, placebo-controlled, parallel-group study of the efficacy, safety, and tolerability of THC:CBD extract and THC extract in patients with intractable cancer-related pain. J Pain Symptom Manage. (2010) 39:167–79. 10.1016/j.jpainsymman.2009.06.008 19896326

[22] Langford RM Mares J Novotna A Vachova M Novakova I Notcutt W. A double-blind, randomized, placebo-controlled, parallel-group study of THC/CBD oromucosal spray in combination with the existing treatment regimen, in the relief of central neuropathic pain in patients with multiple sclerosis. J Neurol. (2013) 260:984–97. 10.1007/s00415-012-6739-4 23180178

[23] De Vries M Van Rijckevorsel DC Wilder-Smith OH Van Goor H. Dronabinol and chronic pain: importance of mechanistic considerations. Exp Opin Pharmacother. (2014) 15:1525–34. 10.1517/14656566.2014.918102 24819592

[24] Schimrigk S Marziniak M Neubauer C Kugler EM Werner G Abramov-Sommariva D. Dronabinol is a safe long-term treatment option for neuropathic pain patients. Eur Neurol. (2017) 78:320–9. 10.1159/000481089 29073592 PMC5804828

[25] Boehnke KF Clauw DJ. Brief commentary: cannabinoid dosing for chronic pain management. Ann Intern Med. (2019) 170:118. 10.7326/M18-2972 30615778

[26] Bhaskar A Bell A Boivin M Briques W Brown M Clarke H. Consensus recommendations on dosing and administration of medical cannabis to treat chronic pain: results of a modified Delphi process. J Cannabis Res. (2021) 3:22. 10.1186/s42238-021-00073-1 34215346 PMC8252988

[27] Hill KP Abrams DI. A cannabis oracle? Delphi method not a substitute for randomized controlled trials of cannabinoids as therapeutics. J Cannabis Res. (2021) 3:23. 10.1186/s42238-021-00074-0 34215325 PMC8254257

[28] O'Connor CM Ring D. Correlation of single assessment numeric evaluation (SANE) with other patient reported outcome measures (PROMs). Arch Bone Joint Surg. (2019) 7:303–6. 31448305 PMC6686068

[29] Zuardi AW Shirakawa I Finkelfarb E Karniol IG. Action of cannabidiol on the anxiety and other effects produced by delta 9-THC in normal subjects. Psychopharmacology. (1982) 76:245–50. 10.1007/BF00432554 6285406

[30] Tumati S Lanctôt KL Wang R Li A Davis A Herrmann N. Medical cannabis use among older adults in Canada: self-reported data on types and amount used, perceived effects. Drugs Aging. (2021) 39:153–63. 10.1007/s40266-021-00913-y 34940961 PMC8696251

[31] Mlost J Bryk M Starowicz K. Cannabidiol for pain treatment: focus on pharmacology and mechanism of action. Int J Mol Sci. (2020) 21:1–22. 10.3390/ijms21228870 33238607 PMC7700528

[32] Urits I Gress K Charipova K Habib K Lee D Lee C. Use of cannabidiol (CBD) for the treatment of chronic pain. Best Pract Res Clin Anaesthesiol. (2020) 34:463–77. 10.1016/j.bpa.2020.06.004 33004159

[33] Häuser W Fitzcharles MA. The perils of overestimating the efficacy of cannabis-based medicines for chronic pain management. Pain Phys. (2018) 21:E79–85. 10.36076/ppj.2018.1.E79 29357344

[34] De Vita MJ Maisto SA Gilmour CE McGuire L Tarvin E Moskal D. The effects of cannabidiol and analgesic expectancies on experimental pain reactivity in healthy adults: a balanced placebo design trial. Exp Clin Psychopharmacol. (2021). 10.1037/pha0000465. [Epub ahead of print]. 34251840 PMC8531169

[35] Spinella TC Stewart SH Naugler J Yakovenko I Barrett SP. Evaluating cannabidiol (CBD) expectancy effects on acute stress and anxiety in healthy adults: a randomized crossover study. Psychopharmacology. (2021) 238:1965–77. 10.1007/s00213-021-05823-w 33813611 PMC8233292

[36] Klinger R Colloca L Bingel U Flor H. Placebo analgesia: clinical applications. Pain. (2014) 155:1055–8. 10.1016/j.pain.2013.12.007 24333780 PMC4011974

[37] McLean CP Asnaani A Litz BT Hofmann SG. Gender differences in anxiety disorders: prevalence, course of illness, comorbidity and burden of illness. J Psychiatr Res. (2011) 45:1027–35. 10.1016/j.jpsychires.2011.03.006 21439576 PMC3135672

[38] Gruber SA Smith RT Dahlgren MK Lambros AM Sagar KA. No pain, all gain? Interim analyses from a longitudinal, observational study examining the impact of medical cannabis treatment on chronic pain and related symptoms. Exp Clin Psychopharmacol. (2021) 29:147–56. 10.1037/pha0000435 33764103

[39] Bonn-Miller MO Loflin MJE Thomas BF Marcu JP Hyke T Vandrey R. Labeling accuracy of cannabidiol extracts sold online. JAMA. (2017) 318:1708–9. 10.1001/jama.2017.11909 29114823 PMC5818782

[40] Zuardi AW Hallak JEC Crippa JAS. Interaction between cannabidiol (CBD) and Δ(9)-tetrahydrocannabinol (THC): influence of administration interval and dose ratio between the cannabinoids. Psychopharmacology. (2012) 219:247–9. 10.1007/s00213-011-2495-x 21947314

[41] Wade DT Makela P Robson P House H Bateman C. Do cannabis-based medicinal extracts have general or specific effects on symptoms in multiple sclerosis? A double-blind, randomized, placebo-controlled study on 160 patients. Multiple Scler. (2004) 10:434–41. 10.1191/1352458504ms1082oa 15327042

[42] Aviram J Lewitus GM Vysotski Y Berman P Shapira A Procaccia S. Sex differences in medical cannabis-related adverse effects. Pain. (2021). 10.1097/j.pain.0000000000002463. [Epub ahead of print]. 34538843 PMC9009319

