# Supplementary material for: Sex-Dependent Prescription Patterns and Clinical Outcomes Associated With the Use of Two Oral Cannabis Formulations in the Multimodal Management of Chronic Pain Patients in Colombia
Source: Front Pain Res (Lausanne). 2022 Mar 24;3:854795. doi: 10.3389/fpain.2022.854795 (PMC8987276; doi:10.3389/fpain.2022.854795)
Supplement: Supplementary file 1 [file Data_Sheet_1.PDF]

Líder en la confiabilidad de sus análisis

Informe Analítico

No. 32756

Bogotá, 2021-12-06

**Producto:**	Preparación Magistral de Cannabis Sativa 2.0% P/V Rico en THC Variedad TA-3-008	**Lote No.:**	2EE-10-TA-N-0011260821-6
**Presentación:**	Frasco x 30mL	**Forma Farmacéutica:**	Solucion Oral
**Cliente:**	BIOVIE S.A.S.	**Tamaño del Lote:**	N.A
**Dirección:**	Cll 94 No. 58 - 40	**Procedencia:**	BIOVIE S.A.S.
**Fecha de Ingreso:**	2021-09-01	**Cotización No.:**	2020-3068
**Muestra No.:**	149702	**Fecha de Expiración:**	2021-10-25
**Fecha de Fabricación:**	2021-08-26	**Fecha de Análisis:**	2021-12-06
**Tipo de Análisis:**	Fisicoquímico		

MÉTODO	ENSAYO	ESPECIFICACIÓN	RESULTADO	CONCEPTO
Quasfar	Aspecto	Líquido aceitoso, viscoso, color á mbar con aroma herbal caracterí stico a Cannabis.	Resultado: Cumple<br>Observación: Líquido aceitoso, viscoso, color ámbar con aroma herbal caracterí stico a Cannabis	Cumple
Quasfar	Identificación de Cannabinoides (HPLC)	El tiempo de retención del pico principal en el cromatograma de la solución de prueba corresponde con el del cromatograma de la solución está ndar según se obtiene en la valoración	Identificación THC-d9: Cumple<br>Identificación THCA: Cumple	Cumple
Quasfar	Valoración de THC Total (UHPLC)	Contenido Total de THC: (THCd9 + THCA x 0.877) 2.0% P/V<br>No menos del 90% y no más del 110% del valor declarado	M1 THCd9 (Conc): 1.81 % p/v<br>M1 THCd9 (%): 92.60 %<br>M2 THCd9 (Conc): 1.81 % p/v<br>M2 CTHCd9 (%): 92.30 %<br>Promedio THCd9: 1.81 % p/v<br>Promedio THCd9 (%): 92.50 %<br>RSD THCd9 (%): 0.2 %<br>Promedio THCA: 0.04 % p/v<br>Promedio THCA (%): 0.00 %<br>Total THC (Conc): 1.85 % p/v<br>Total THC (%): 92.50 %	Cumple
Quasfar	Valoración de CBD Total (UHPLC)	Contenido Total de CBD (CBD + CBDA x 0.877) < 1.0% P/V	Promedio CBD: 0.00 % p/v<br>Promedio CBDA: 0.00 % p/v<br>Total CBD (Conc): 0.00 % p/v	Cumple
Quasfar	Valoración de CBN (UHPLC)	NMT 1.0% CBN P/V	Promedio CBN: 0.00 % p/v	Cumple

Av. Cra. 68 No. 17-90 Piso 2
PBX: 447 0047
Bogotá, D.C. - Colombia
www.quasfar.com.co

Concepto Final: LA MUESTRA ANALIZADA CUMPLE CON LAS ESPECIFICACIONES DEL LABORATORIO PROPIETARIO PARA LOS ENSAYOS REALIZADOS.

Anexos:

Se adjuntan las hojas de ruta (hojas de datos primarios de los análisis) las cuales hacen parte integral del presente informe en las que se puede verificar la trazabilidad de los estándares, reactivos, medios de cultivo, materiales de vidrio, equipos usados en cada uno de los análisis, así como las fechas de inicio de incubación y lecturas de los aná lisis microbiológicos; en caso que aplique (n).

Analista(s) Responsable(s): EMURCIA

Revisó / Aprobó:

Mercedes Ef de Cuadros

Mercedes Ferrer

Director Técnico y de Estudios

Nuestros procesos analíticos están respaldados por Labware LIMS, sistema de información que sigue estricto cumplimiento de datos primarios, trazabilidad, integridad y seguridad de la información, cumpliendo con FDA CFR 21 parte 11.

Este certificado no puede ser reproducido sin previa autorización escrita de la Dirección Técnica de Quasfar M&F S.A.
Los resultados son válidos para la muestra analizada y no para otra de la misma procedencia.

Av. Cra. 68 No. 17-90 Piso 2
PBX: 447 0047
Bogotá, D.C. - Colombia
www.quasfar.com.co

HOJA DE CALCULO QUASFAR M&F S.A.
CONTENIDO DE FITOCANNABINOIDES

**Nombre Del Producto:**	CANNABIS PREPARACIÓN MAGISTRAL THC 2.0% ACEITE
**Tipo De Muestra:**	FISICOQUÍMICO
**Lote:**	2EE-10-TA-N-0011260821-6
**Muestra:**	149702

ESTANDAR - CURVA DE CALIBRACION

FITOCANNABIDOIDES	Ácido Cannabidiólico (CBDA)		Cannabinol (CBN)		9-Tetrahidrocannabinol (Δ9-THC)		Ácido Tetrahidrocannabinolico A (THCA-A)		Cannabidiol (CBD)	
Alicuota del Estandar / 2mL	Concentracion µg/mL	Area (Astd)	Concentracion µg/mL	Area (Astd)	Concentracion µg/mL	Area (Astd)	Concentracion µg/mL	Area (Astd)	Concentracion µg/mL	Area (Astd)
0.02 mL (SM) /1mL (P1)	5.00	16.74555	5.00	26.72388	5.00	10.52096	5.01	15.14045	5.01	11.60611
0.02 mL (SM) /1mL (P1)	5.00	16.78599	5.00	26.47970	5.00	10.24760	5.01	14.97855	5.01	11.56633
0.02 mL (SM) /1mL (P1)	5.00	16.91982	5.00	26.55656	5.00	10.96090	5.01	15.18813	5.01	12.08453
0.06 mL (SM) /1mL (P2)	15.00	53.36622	15.00	74.59605	15.00	30.59950	15.02	48.84549	15.03	32.00980
0.06 mL (SM) /1mL (P2)	15.00	53.20448	15.00	74.15126	15.00	30.81016	15.02	48.22856	15.03	32.51368
0.06 mL (SM) /1mL (P2)	15.00	53.55395	15.00	73.51779	15.00	30.70448	15.02	48.60644	15.03	32.50823
0.1 mL (SM) /1mL (P3)	25.00	97.28935	25.00	137.46196	25.00	57.41228	25.03	90.61348	25.05	59.08337
0.1 mL (SM) /1mL (P3)	25.00	97.39140	25.00	137.55332	25.00	57.43350	25.03	90.19426	25.05	58.84093
0.1 mL (SM) /1mL (P3)	25.00	97.76012	25.00	137.42469	25.00	56.94273	25.03	88.89687	25.05	59.14621
0.2 mL (SM) /1mL (P4)	50.00	187.06599	50.00	301.20492	50.00	127.29305	50.05	173.98296	50.10	128.98622
0.2 mL (SM) /1mL (P4)	50.00	190.91350	50.00	301.78294	50.00	129.23495	50.05	173.71441	50.10	130.54738
0.2 mL (SM) /1mL (P4)	50.00	190.37885	50.00	303.27756	50.00	129.66141	50.05	175.14476	50.10	130.12084
0.3 mL (SM) /1mL (P5)	75.00	296.55476	75.00	435.30587	75.00	184.90332	75.08	274.56484	75.14	186.78770
0.3 mL (SM) /1mL (P5)	75.00	297.68248	75.00	435.27860	75.00	186.21023	75.08	277.57135	75.14	186.56741
0.3 mL (SM) /1mL (P5)	75.00	297.15025	75.00	434.45975	75.00	184.39475	75.08	276.94707	75.14	185.71806
0.4 mL (SM) /1mL (P6)	100.00	372.74148	100.00	577.52297	100.00	245.62424	100.10	346.92630	100.19	246.19951
0.4 mL (SM) /1mL (P6)	100.00	376.49793	100.00	576.11177	100.00	245.32159	100.10	350.75291	100.19	245.70387
0.4 mL (SM) /1mL (P6)	100.00	373.40731	100.00	576.78087	100.00	244.72860	100.10	347.71911	100.19	246.19552
**Intersecciòn (b)**	**-0.8330**		**-5.7352**		**-3.3593**		**-1.9662**		**-1.7722**	
**Pendiente (m)**	**3.8277**		**5.8758**		**2.5105**		**3.5685**		**2.4991**	
**Coef. Correlaciòn (r)**	**0.9990**		**0.9993**		**0.9992**		**0.9990**		**0.9992**	
**Coef. Determinaciòn (r2)**	**0.9980**		**0.9987**		**0.9984**		**0.9979**		**0.9985**	

MUESTRA 1 - CONCENTRADA

Activo	Area (Am)		Promedio	RSD ≤ 10%	Concentracion mg/mL	%
Lectura	1	2				
THCA-A	23.66734	24.83757	24.2525	3.4	0.45	0.04
Cannabidiol (CBD)	No detectado	No detectado	N.A	N.A	N.A	No detectado
Ácido Cannabidiólico (CBDA)	No detectado	No detectado	N.A	N.A	N.A	No detectado
Cannabinol (CBN)	No detectado	No detectado	N.A	N.A	N.A	No detectado

MUESTRA 1

Activo	Area (Am)		Promedio	RSD ≤ 10%	Concentracion mg/mL	% THC	% THC Total	% (Cant Etiq)
Lectura	1	2						
9-Tetrahidrocannabinol (Δ9-THC)	123.63198	124.14604	123.88901	0.3	18.14	1.81	**1.85**	**92.6**

MUESTRA 2

Activo	Area (Am)		Promedio	RSD ≤ 10%	Concentracion mg/mL	% THC	% THC Total	% (Cant Etiq)
Lectura	1	2						
9-Tetrahidrocannabinol (Δ9-THC)	122.39606	125.18356	123.78981	1.6	18.07	1.81	**1.85**	**92.3**

I.L.C. Inferior al límite de cuantificación

MUESTRA	Concentracion mg/mL	% THC	% THC Total
**mg/g 9-Tetrahidrocannabinol (Δ9-THC) Promedio (M1-M2-M3)**	18.11	**1.81**	1.85
**% 9-Tetrahidrocannabinol (Δ9-THC) (Respecto a lo etiquetado)**	**92.5**		
**RSD**	**0.24**		
**Cannabinol (CBN)**	**N.A**	**No detectado**	
**THCA-A**	**0.45**	**0.04**	
**Ácido Cannabidiólico (CBDA)**	**N.A**	**No detectado**	

DATOS

Fitocannabinoides - Potencia estándar / Concentracion estándar	(µg/mL)
**Estándar Ácido Cannabidiólico (CBDA) Lote:**	**0626266**
**Estándar Cannabinol (CBN) Lote:**	**0584229**
**Estándar 9-Tetrahidrocannabinol (Δ9-THC) Lote:**	**0624020**
**Estándar Ácido Tetrahidrocannabinolico A (THCA-A) Lote:**	**0626035**
**Estándar Cannabidiol (CBD) Lote:**	**A0172221**
Ácido Cannabidiólico (CBDA)	1000
Cannabinol (CBN)	1000
Δ9-Tetrahidrocannabinol (Δ9-THC)	1000
Ácido A-Tetrahidrocannabinólico (THCA-A)	1001
Cannabidiol (CBD)	1001.9
Factor de Dilución Muestra - Concentrada (10 mL) (Dm)	10.0
Factor de Dilución Muestra - Diluida ((10x5)/0.85) (Dm)	58.8
Peso de Muestra 1 (Pm) mg	149.4
Peso de Muestra 2 (Pm) mg	149.8
Densidad (g/mL) (d)	0.9088
Cantidad Etiquetada (g/100mL) (Cant Etiq) (%) (9-Tetrahidrocannabinol (Δ9-THC))	2.0

CALCULOS

CALCULOS
Concentración (mg fitocannabinoides/mL) = ((Am - b)*Dm*d) / (m*Pm)
% = (Concentración (mg/mL) x 100 mL) / 1000
% (Respecto a lo etiquetado) = (%*100)/Cant Etiq

Analista

Leidy Ibagon Franco

2021-12-01
