# Supplementary material for: Sex-Dependent Prescription Patterns and Clinical Outcomes Associated With the Use of Two Oral Cannabis Formulations in the Multimodal Management of Chronic Pain Patients in Colombia
Source: Front Pain Res (Lausanne). 2022 Mar 24;3:854795. doi: 10.3389/fpain.2022.854795 (PMC8987276; doi:10.3389/fpain.2022.854795)
Supplement: Supplementary file 2 [file Data_Sheet_2.PDF]

Líder en la confiabilidad de sus análisis

Informe Analítico

No. 11259

Bogotá, 2020-07-02

QUASFAR M&F S.A.

**Producto:**	Preparación Magistral de Cannabis Sativa 14 mg/mL en CBD y 12 mg/mL en THC Variedad WW-3-011	**Lote No.:**	1.2EE-10-WW-N-001-260520-1-16
**Presentación:**	Frasco 30mL	**Forma Farmacéutica:**	Solucion Oral
**Cliente:**	BIOVIE S.A.S.	**Tamaño del Lote:**	N.E.
**Dirección:**	Cll 94 No. 58 - 40	**Procedencia:**	BIOVIE S.A.S.
**Fecha de Ingreso:**	2020-06-05	**Cotización No.:**	2020-3068
**Muestra No.:**	126244	**Fecha de Expiración:**	2020-06-25
**Fecha de Fabricación:**	2020-05-26	**Fecha de Análisis:**	2020-06-25
**Tipo de Análisis:**	Fisicoquímico/Microbiológico		

MÉTODO	ENSAYO	ESPECIFICACIÓN	RESULTADO	CONCEPTO
Quasfar	Aspecto	Líquido aceitoso, viscoso, color á mbar con aroma herbal caracterí stico a Cannabis.	Resultado: Cumple<br>Observación: Líquido aceitoso, viscoso, color ámbar con aroma herbal caracterí stico a Cannabis.	Cumple
Quasfar	Identificación de Cannabinoides (HPLC)	El tiempo de retención del pico principal en el cromatograma de la solución de prueba corresponde con el del cromatograma de la solución está ndar según se obtiene en la valoración	Identificación THC-d9: Cumple<br>Identificación CBD: Cumple<br>Identificación CBDA: Cumple<br>Identificación CBN: Cumple	Cumple
Quasfar	Valoración de THC Total (UHPLC)	Contenido Total de THC: (THCd9 + THCA x 0.877) 1.2% P/V<br>No menos del 90% y no más del 110% del valor declarado	M1 THCd9 (Conc): 1.23 % p/v<br>M1 THCd9 (%): 102.20 %<br>M2 THCd9 (Conc): 1.21 % p/v<br>M2 CTHCd9 (%): 100.50 %<br>Promedio THCd9: 1.22 % p/v<br>Promedio THCd9 (%): 101.40 %<br>RSD THCd9 (%): 1.2 %<br>Promedio THCA: 0.00 % p/v<br>Promedio THCA (%): 0.00 %<br>Total THC (Conc): 1.22 % p/v<br>Total THC (%): 101.40 %	Cumple

MÉTODO	ENSAYO	ESPECIFICACIÓN	RESULTADO	CONCEPTO
Quasfar	Valoración de CBD Total (UHPLC)	Contenido Total de CBD (CBD + CBDA x 0.877) 1.4 % P/V<br>No menos del 90% y no más del 110% del valor declarado	M1 CBD (Conc): 1.38 % p/v<br>M1 CBD (%): 98.80 %<br>M2 CBD (Conc): 1.38 % p/v<br>M2 CBD (%): 98.90 %<br>Promedio CBD: 1.38 % p/v<br>Promedio CBD (%): 98.90 %<br>RSD CBD (%): 0.1 %<br>Promedio CBDA: 0.05 % p/v<br>Promedio CBDA (%): 0.05 %<br>Total CBD (Conc): 1.42 % p/v<br>Total CBD (%): 98.94 %	Cumple
Quasfar	Valoración de CBN (UHPLC)	NMT 1.0 % de CBN	Promedio CBN: 0.00 % p/v	Cumple
USP Vigente	Mesófilos aerobios	<10^3 UFC/g	Resultado: <10 UFC/g	Cumple
USP Vigente	Hongos y Levaduras	<10^2 UFC/g	Resultado: <10 UFC/g	Cumple
USP Vigente	E.coli	Ausencia	Conclusión: Ausente	Cumple
USP Vigente	Salmonella sp	Ausencia	Conclusión: Ausente	Cumple
USP Vigente	Bacterias gram negativas tolerantes a bilis	<10^2 UFC/g	Resultado: <10 UFC/g	Cumple

Concepto Final: LA MUESTRA ANALIZADA CUMPLE CON LAS ESPECIFICACIONES DEL LABORATORIO PROPIETARIO PARA LOS ENSAYOS REALIZADOS.

Anexos:

Se adjuntan las hojas de ruta (hojas de datos primarios de los análisis) las cuales hacen parte integral del presente informe en las que se puede verificar la trazabilidad de los estándares, reactivos, medios de cultivo, materiales de vidrio, equipos usados en cada uno de los análisis, así como las fechas de inicio de incubación y lecturas de los an álisis microbiológicos; en caso que aplique (n).

Av. Cra. 68 No. 17-90 Piso 2
PBX: 447 0047
Bogotá, D.C. - Colombia
www.quasfar.com.co

Analista(s) Responsable(s) (código): 74, 117

Revisó / Aprobó:

Mercedes Ferrer

Director Técnico y de Estudios

Nuestros procesos analíticos están respaldados por Labware LIMS, sistema de información que sigue estricto cumplimiento, trazabilidad, integridad y seguridad de la información, cumpliendo con FDA CFR 21 parte 11.

Este certificado no puede ser reproducido sin previa autorización escrita de la Dirección Técnica de Quasfar M&F S.A.
Los resultados son válidos para la muestra analizada y no para otra de la misma procedencia.

Av. Cra. 68 No. 17-90 Piso 2
PBX: 447 0047
Bogotá, D.C. - Colombia
www.quasfar.com.co

Código: VD-005

HOJA DE CALCULO QUASFAR M&F S.A.
CONTENIDO DE FITOCANNABINOIDES

Revisión: 01

**Nombre Del Producto:**	PREPARACION MAGISTRA DE CANNABIS SATIVA 14 mg/mL EN CBD Y 12 mg/mL THC
**Tipo De Muestra:**	PREPARACION MAGISTRAL
**Lote:**	1.2EE-10-WW-N-001-260520-1-16
**Muestra:**	126244

ESTANDAR - CURVA DE CALIBRACION										
FITOCANNABIDOIDES	Ácido Cannabidiólico (CBDA)		Cannabinol (CBN)		9-Tetrahidrocannabinol (Δ9-THC)		Ácido Tetrahidrocannabinolico A (THCA-A)		Cannabidiol (CBD)	
Alicuota del Estandar / 0.3mL	Concentracion µg/mL	Area (Astd)	Concentracion µg/mL	Area (Astd)	Concentracion µg/mL	Area (Astd)	Concentracion µg/mL	Area (Astd)	Concentracion µg/mL	Area (Astd)
0.010	8.33	21248	8.33	17997	8.33	7984	8.33	20522	8.33	12750
0.010	8.33	21317	8.33	18442	8.33	7785	8.33	19606	8.33	13389
0.010	8.33	20740	8.33	17739	8.33	7985	8.33	18973	8.33	12298
0.030	25.00	64096	25.00	58873	25.00	23958	25.00	56105	25.00	30666
0.030	25.00	63621	25.00	57610	25.00	23320	25.00	54615	25.00	29756
0.030	25.00	63751	25.00	57905	25.00	24772	25.00	56979	25.00	31506
0.060	50.00	124748	50.00	133174	50.00	55039	50.00	110770	50.00	61596
0.060	50.00	122923	50.00	132945	50.00	53983	50.00	108403	50.00	62067
0.060	50.00	123720	50.00	134354	50.00	53592	50.00	107646	50.00	61702
0.140	116.67	271687	116.67	293119	116.67	119949	116.67	245205	116.67	133129
0.140	116.67	273486	116.67	294754	116.67	119173	116.67	243214	116.67	135109
0.140	116.67	268393	116.67	296534	116.67	120725	116.67	241855	116.67	134401
0.280	233.33	599775	233.33	637403	233.33	260074	233.33	538073	233.33	287007
0.280	233.33	592094	233.33	641101	233.33	261930	233.33	536080	233.33	286107
0.280	233.33	584906	233.33	638779	233.33	259582	233.33	527407	233.33	286951
**Intersecciòn (b)**	**-3869.3567**		**-9680.3724**		**-3698.9945**		**-4704.2976**		**-10.4314**	
**Pendiente (m)**	**2518.8810**		**2750.7189**		**1119.4892**		**2273.8596**		**1214.3004**	
**Coef. Correlaciòn (r)**	**0.9988**		**0.9993**		**0.9992**		**0.9988**		**0.9992**	
**Coef. Determinaciòn (r2)**	**0.9976**		**0.9985**		**0.9984**		**0.9975**		**0.9985**	

MUESTRA 1 - CONCENTRADA						
**Activo**	**Area (Am)**		**Promedio**	**RSD ≤ 10%**	**Concentracion mg/mL**	**%**
**Lectura**	**1**	**2**				
THCA-A	No detectado	No detectado	N.A	N.A	N.A	No detectado
Ácido Cannabidiólico (CBDA)	61142	62052	61597	1.0	0.47	0.05
Cannabinol (CBN)	17444	17641	17543	0.8	I.L.C	I.L.C

MUESTRA 1							
**Activo**	**Area (Am)**		**Promedio**	**RSD ≤ 10%**	**Concentracion mg/mL**	**%**	**% (Cant Etiq)**
**Lectura**	**1**	**2**					
9-Tetrahidrocannabinol (Δ9-THC)	60780	59985	60383	0.9	12.27	1.23	**102.2**

MUESTRA 2							
**Activo**	**Area (Am)**		**Promedio**	**RSD ≤ 10%**	**Concentracion mg/mL**	**%**	**% (Cant Etiq)**
**Lectura**	**1**	**2**					
9-Tetrahidrocannabinol (Δ9-THC)	59252	59706	59479	0.5	12.06	1.21	**100.5**

I.L.C. Inferior al límite de cuantificación

**MUESTRA**	**Concentracion mg/mL**	**%**
**mg/g 9-Tetrahidrocannabinol (Δ9-THC) Promedio (M1-M2-M3)**	12.16	**1.22**
**% 9-Tetrahidrocannabinol (Δ9-THC) (Respecto a lo etiquetado)**	**101.4**	
**RSD**	**1.19**	
**Cannabinol (CBN)**	**I.L.C**	**I.L.C**
**THCA-A**	N.A	No detectado
**Ácido Cannabidiólico (CBDA)**	0.47	0.05

Cannabidiol(CBD)							
**MUESTRA 1**							
**Activo**	**Area (Am)**		**Promedio**	**RSD ≤ 10%**	**Concentracion mg/mL**	**%**	**% (Cant Etiq)**
**Lectura**	**1**	**2**					
Cannabidiol (CBD)	77900	78848	78374	0.9	13.83	1.38	**98.8**

MUESTRA 2							
**Activo**	**Area (Am)**		**Promedio**	**RSD ≤ 10%**	**Concentracion mg/mL**	**%**	**% (Cant Etiq)**
**Lectura**	**1**	**2**					
Cannabidiol (CBD)	79100	78216	78658	0.8	13.85	1.38	**98.9**

**MUESTRA**	**Concentracion mg/mL**	**%**
**mg/g Cannabidiol (CBD) Promedio (M1-M2-M3)**	13.84	**1.38**
**% Cannabidiol (CBD) (Respecto a lo etiquetado)**	**98.9**	
**RSD**	**0.07**	

DATOS	
**Fitocannabinoides - Potencia estándar / Concentracion estándar**	**(µg/mL)**
**Estándar Ácido Cannabidiólico (CBDA) Lote:**	**0557556**
**Estándar Cannabinol (CBN) Lote:**	**0580104**
**Estándar 9-Tetrahidrocannabinol (Δ9-THC) Lote:**	**0580104**
**Estándar Ácido Tetrahidrocannabinolico A (THCA-A) Lote:**	**0558070**
**Estándar Cannabidiol (CBD) Lote:**	**0580104**
Ácido Cannabidiólico (CBDA)	1000.0
Cannabinol (CBN)	1000.0
Δ9-Tetrahidrocannabinol (Δ9-THC)	1000.0
Ácido A-Tetrahidrocannabinólico (THCA-A)	1000.0
Cannabidiol (CBD)	1000.0
Factor de Dilución Muestra - Concentrada (10 mL) (Dm)	10.0
Factor de Dilución Muestra - Diluida ((10x10)/0.85) (Dm)	117.6
Peso de Muestra 1 (Pm) mg	500.3
Peso de Muestra 2 (Pm) mg	501.6
Densidad (g/mL) (d)	0.9113
Cantidad Etiquetada (g/100mL) (Cant Etiq) (%) (9-Tetrahidrocannabinol (Δ9-THC))	1.2
Cantidad Etiquetada (g/100mL) (Cant Etiq) (%) (Cannabidiol (CBD))	1.4

CALCULOS
Concentración (mg fitocannabinoides/mL) = ((Am - b)*Dm*d) / (m*Pm)
% = (Concentración (mg/mL) x 100 mL) / 1000
% (Respecto a lo etiquetado) = (%*100)/Cant Etiq

Analista

Edinson Murcia Riaño

2020-06-16
