# Supplementary material for: Sex-Dependent Prescription Patterns and Clinical Outcomes Associated With the Use of Two Oral Cannabis Formulations in the Multimodal Management of Chronic Pain Patients in Colombia
Source: Front Pain Res (Lausanne). 2022 Mar 24;3:854795. doi: 10.3389/fpain.2022.854795 (PMC8987276; doi:10.3389/fpain.2022.854795)
Supplement: Supplementary file 3 [file Data_Sheet_3.PDF]

**Líder en la confiabilidad de sus análisis**

**Informe Analítico**

**No. 21826**

**Bogotá, 2021-04-29**

QUASFAR M&F S.A.

| | | | |
|---|---|---|---|
| **Producto:** | Preparación Magistral de Cannabis Sativa 3.0% P/V Rico en CBD Variedad FT-1-009 | **Lote No.:** | EE-7.3-FT-A-0011160421-1 |
| **Presentación:** | Frasco x 30mL | **Forma Farmacéutica:** | Solucion Oral |
| **Cliente:** | BIOVIE S.A.S. | **Tamaño del Lote:** | N.E. |
| **Dirección:** | Cll 94 No. 58 - 40 | **Procedencia:** | BIOVIE S.A.S. |
| **Fecha de Ingreso:** | 2021-04-20 | **Cotización No.:** | 2020-3068 |
| **Muestra No.:** | 141426 | **Fecha de Expiración:** | 2021-06-15 |
| **Fecha de Fabricación:** | 2021-04-16 | **Fecha de Análisis:** | 2021-04-27 |
| **Tipo de Análisis:** | Fisicoquímico | | |

| MÉTODO | ENSAYO | ESPECIFICACIÓN | RESULTADO | CONCEPTO |
|---|---|---|---|---|
| Quasfar | Aspecto | Líquido aceitoso, viscoso, color á mbar con aroma herbal caracterí stico a Cannabis. | Resultado: Cumple<br>Observación: Líquido aceitoso, viscoso, color ámbar con aroma herbal caracterí stico a Cannabis | Cumple |
| Quasfar | Identificación de Cannabinoides (HPLC) | El tiempo de retención del pico principal en el cromatograma de la solución de prueba corresponde con el del cromatograma de la solución está ndar según se obtiene en la valoración. | Identificación THC-d9: Cumple<br>Identificación CBD: Cumple | Cumple |
| Quasfar | Valoración de CBD Total ( UHPLC) | Contenido Total CBD: (CBD+ CBDAx0.877) es de 3.0% P/V No menos del 90% y no más del 110% del valor declarado | M1 CBD (Conc): 3.08 % p/v<br>M1 CBD (%): 102.70 %<br>M2 CBD (Conc): 3.08 % p/v<br>M2 CBD (%): 102.50 %<br>Promedio CBD: 3.08 % p/v<br>Promedio CBD (%): 102.60 %<br>RSD CBD (%): 0.1 %<br>Promedio CBDA: 0.00 % p/v<br>Promedio CBDA (%): 0.00 %<br>Total CBD (Conc): 3.08 % p/v<br>Total CBD (%): 102.60 % | Cumple |
| Quasfar | Valoración de THC Total ( UHPLC) | Contenido Total de THC (THC + THCA x 0.877) < 0.2% P/V | Promedio THCd9: 0.00 % p/v<br>Promedio THCA: 0.00 % p/v<br>Total THC (Conc): 0.00 % p/v | Cumple |
| Quasfar | Valoración de CBN ( UHPLC) | NMT 1.0% P/V CBN | Promedio CBN: 0.00 % p/v | Cumple |

Av. Cra. 68 No. 17-90 Piso 2
PBX: 447 0047
Bogotá, D.C. - Colombia
www.quasfar.com.co

**Concepto Final:** LA MUESTRA ANALIZADA CUMPLE CON LAS ESPECIFICACIONES DEL LABORATORIO PROPIETARIO PARA LOS ENSAYOS REALIZADOS.

**Anexos:**

Se adjuntan las hojas de ruta (hojas de datos primarios de los análisis) las cuales hacen parte integral del presente informe en las que se puede verificar la trazabilidad de los estándares, reactivos, medios de cultivo, materiales de vidrio, equipos usados en cada uno de los análisis, así como las fechas de inicio de incubación y lecturas de los ana lisis microbiológicos; en caso que aplique (n).

**Analista(s) Responsable(s) (código):** 74

**Revisó / Aprobó:**

**Mercedes Ferrer**

**Director Técnico y de Estudios**

**Nuestros procesos analíticos están respaldados por Labware LIMS, sistema de información que sigue estricto cumplimiento, trazabilidad, integridad y seguridad de la información, cumpliendo con FDA CFR 21 parte 11.**

**Este certificado no puede ser reproducido sin previa autorización escrita de la Dirección Técnica de Quasfar M&F S.A.**
**Los resultados son válidos para la muestra analizada y no para otra de la misma procedencia.**

Av. Cra. 68 No. 17-90 Piso 2
PBX: 447 0047
Bogotá, D.C. - Colombia
www.quasfar.com.co

**Código: VD-005**

## HOJA DE CALCULO QUASFAR M&F S.A.
## CONTENIDO DE FITOCANNABINOIDES

**Revisión: 01**

**Nombre Del Producto:** CANNABIS PREPARACION MAGISTRAL CBD 3%
**Tipo De Muestra:** Preparación Magistral
**Lote:** EE-7,3-FT-A-0011160421-1
**Muestra:** 141426

| ESTANDAR - CURVA DE CALIBRACION | | | | | | | | | | |
|---|---|---|---|---|---|---|---|---|---|---|
| **FITOCANNABIDOIDES** | **Ácido Cannabidiólico (CBDA)** | | **Cannabinol (CBN)** | | **9-Tetrahidrocannabinol (Δ9-THC)** | | **Ácido Tetrahidrocannabinolico A (THCA-A)** | | **Cannabidiol (CBD)** | |
| Alicuota del Estandar / 0,3mL | Concentracion µg/mL | Area (Astd) | Concentracion µg/mL | Area (Astd) | Concentracion µg/mL | Area (Astd) | Concentracion µg/mL | Area (Astd) | Concentracion µg/mL | Area (Astd) |
| 0.010 | 8.33 | 18152 | 8.32 | 26898 | 8.36 | 9721 | 8.31 | 17399 | 8.35 | 10172 |
| 0.010 | 8.33 | 18903 | 8.32 | 26334 | 8.36 | 9793 | 8.31 | 17533 | 8.35 | 10559 |
| 0.010 | 8.33 | 19866 | 8.32 | 28890 | 8.36 | 10834 | 8.31 | 18243 | 8.35 | 11287 |
| 0.030 | 24.99 | 29285 | 24.95 | 38029 | 25.07 | 16926 | 24.94 | 22729 | 25.06 | 16509 |
| 0.030 | 24.99 | 28811 | 24.95 | 35769 | 25.07 | 17048 | 24.94 | 23331 | 25.06 | 14640 |
| 0.030 | 24.99 | 28305 | 24.95 | 36749 | 25.07 | 16104 | 24.94 | 22339 | 25.06 | 15613 |
| 0.060 | 49.97 | 122977 | 49.89 | 163683 | 50.14 | 63299 | 49.87 | 113143 | 50.12 | 70672 |
| 0.060 | 49.97 | 121288 | 49.89 | 162410 | 50.14 | 65663 | 49.87 | 108543 | 50.12 | 68922 |
| 0.060 | 49.97 | 126089 | 49.89 | 160943 | 50.14 | 69856 | 49.87 | 111806 | 50.12 | 70397 |
| 0.140 | 116.60 | 284276 | 116.41 | 384135 | 116.99 | 157069 | 116.36 | 261523 | 116.95 | 160753 |
| 0.140 | 116.60 | 280424 | 116.41 | 373306 | 116.99 | 156918 | 116.36 | 261802 | 116.95 | 159285 |
| 0.140 | 116.60 | 280627 | 116.41 | 375953 | 116.99 | 152824 | 116.36 | 260017 | 116.95 | 157665 |
| 0.280 | 233.19 | 561784 | 232.82 | 752226 | 233.99 | 311796 | 232.73 | 514925 | 233.89 | 316166 |
| 0.280 | 233.19 | 579792 | 232.82 | 771760 | 233.99 | 319236 | 232.73 | 516485 | 233.89 | 317887 |
| 0.280 | 233.19 | 551721 | 232.82 | 737672 | 233.99 | 304794 | 232.73 | 497672 | 233.89 | 309920 |
| **Intersecciòn (b)** | **-11152.4573** | | **-15231.5342** | | **-6624.4350** | | **-10257.8285** | | **-5736.5829** | |
| **Pendiente (m)** | **2478.0921** | | **3317.4754** | | **1366.3646** | | **2253.0560** | | **1378.6111** | |
| **Coef. Correlaciòn ( r)** | **0.9979** | | **0.9979** | | **0.9983** | | **0.9974** | | **0.9979** | |
| **Coef. Determinaciòn (r2)** | **0.9959** | | **0.9957** | | **0.9965** | | **0.9948** | | **0.9957** | |

| MUESTRA 1 - CONCENTRADA | | | | | | |
|---|---|---|---|---|---|---|
| **Activo** | **Area (Am)** | | **Promedio** | **RSD ≤ 10%** | **Concentracion mg/mL** | **%** |
| **Lectura** | **1** | **2** | | | | |
| 9-Tetrahidrocannabinol (Δ9-THC) | 54459 | 49665 | 52062 | 6.5 | 1.30 | 0.13 |
| THCA-A | No detectado | No detectado | N.A | N.A | N.A | No detectado |
| Ácido Cannabidiólico (CBDA) | No detectado | No detectado | N.A | N.A | N.A | 0.00 |
| Cannabinol (CBN) | No detectado | No detectado | N.A | N.A | I.L.C | I.L.C |

| MUESTRA 1 | | | | | | | |
|---|---|---|---|---|---|---|---|
| **Activo** | **Area (Am)** | | **Promedio** | **RSD ≤ 10%** | **Concentracion mg/mL** | **%** | **% (Cant Etiq) (CBD+CBDA)** |
| **Lectura** | **1** | **2** | | | | | |
| Cannabidiol(CBD) | 61481 | 66991 | 64236 | 6.1 | 30.81 | 3.08 | **102.7** |

| MUESTRA 2 | | | | | | | |
|---|---|---|---|---|---|---|---|
| **Activo** | **Area (Am)** | | **Promedio** | **RSD ≤ 10%** | **Concentracion mg/mL** | **%** | **% (Cant Etiq) (CBD+CBDA)** |
| **Lectura** | **1** | **2** | | | | | |
| Cannabidiol(CBD) | 63775 | 63404 | 63590 | 0.4 | 30.75 | 3.08 | **102.5** |

**I.L.C. Inferior al límite de cuantificaciôn**

| **MUESTRA** | **Concentración mg/mL** | **%** |
|---|---|---|
| **mg/mL Cannabidiol(CBD) Promedio (M1-M2)** | 30.78 | **3.08** |
| **% Cannabidiol(CBD) (Respecto a lo etiquetado)** | **102.6** | |
| **RSD** | **0.14** | |
| **Cannabinol (CBN)** | **I.L.C** | **I.L.C** |
| **Ácido Cannabidiólico (CBDA)** | No detectado | No detectado |
| **THCA-A** | N.A | No detectado |
| **9-Tetrahidrocannabinol (Δ9-THC)** | **1.30** | **0.13** |

| DATOS | |
|---|---|
| **Fitocannabinoides - Potencia estándar / Concentracion estándar** | **(µg/mL)** |
| **Estándar Ácido Cannabidiólico (CBDA) Lote:** | **0589171** |
| **Estándar Cannabinol (CBN) Lote:** | |
| **Estándar 9-Tetrahidrocannabinol (Δ9-THC) Lote:** | |
| **Estándar Ácido Tetrahidrocannabinolico A (THCA-A) Lote:** | |
| **Estándar Cannabidiol (CBD) Lote:** | |
| **(±) - Cannabichromene (CBC)** | |
| Ácido Cannabidiólico (CBDA) | 499.7 |
| Cannabinol (CBN) | 498.9 |
| Δ9-Tetrahidrocannabinol (Δ9-THC) | 501.4 |
| Ácido A-Tetrahidrocannabinólico (THCA-A) | 498.7 |
| Cannabidiol (CBD) | 501.2 |
| Factor de Dilución Muestra - Concentrada (10 mL) (Dm) | 10.0 |
| Factor de Dilución Muestra - Diluida ((10x10)/0.5) (Dm) | 200.0 |
| Peso de Muestra 1 (Pm) mg | 302.7 |
| Peso de Muestra 2 (Pm) mg | 300.5 |
| Densidad (d) g/mL | 0.9188 |
| Cantidad Etiquetada (g/100mL) (Cant Etiq) (%) | 3.0 |

| CALCULOS |
|---|
| Concentración (mg fitocannabidoides/mL) = ((Am - b)*Dm*d) / (m*Pm) |
| % = (Concentración (mg/mL) x 100 mL) / 1000 mg |
| % (Respecto a lo etiquetado) = (%*100)/Cant Etiq |

Analista

Edinson Murcia Riaño

**2021-04-23**
