# Supplementary material for: Sex-Dependent Prescription Patterns and Clinical Outcomes Associated With the Use of Two Oral Cannabis Formulations in the Multimodal Management of Chronic Pain Patients in Colombia
Source: Front Pain Res (Lausanne). 2022 Mar 24;3:854795. doi: 10.3389/fpain.2022.854795 (PMC8987276; doi:10.3389/fpain.2022.854795)
Supplement: Supplementary file 4 [file Data_Sheet_4.PDF]

Líder en la confiabilidad de sus análisis

Informe Analítico

No. 11746

Bogotá, 2020-07-17

QUASFAR M&F S.A.

**Producto:**	Preparación Magistral de Cannabis Sativa 10.0% P/V rico en CBD Variedad FT-1-009	**Lote No.:**	15-FT-A-0001230620-1-3
**Presentación:**	Frasco x 50mL	**Forma Farmacéutica:**	Solucion Oral
**Cliente:**	BIOVIE S.A.S.	**Tamaño del Lote:**	N.E.
**Dirección:**	Cll 94 No. 58 - 40	**Procedencia:**	BIOVIE S.A.S.
**Fecha de Ingreso:**	2020-07-07	**Cotización No.:**	2020-3068
**Muestra No.:**	128328	**Fecha de Expiración:**	2020-07-23
**Fecha de Fabricación:**	2020-06-23	**Fecha de Análisis:**	2020-07-16
**Tipo de Análisis:**	Fisicoquímico/Microbiológico		

MÉTODO	ENSAYO	ESPECIFICACIÓN	RESULTADO	CONCEPTO
Quasfar	Aspecto	Líquido aceitoso, viscoso, color á mbar con aroma herbal caracterí stico a Cannabis	Resultado: Cumple<br>Observación: Líquido aceitoso, viscoso, color ámbar con aroma herbal caracterí stico a Cannabis	Cumple
Quasfar	Identificación de Cannabinoides (HPLC)	El tiempo de retención del pico principal en el cromatograma de la solución de prueba corresponde con el del cromatograma de la solución está ndar según se obtiene en la valoración.	Identificación THC-d9: Cumple<br>Identificación CBD: Cumple<br>Identificación CBDA: Cumple<br>Identificación CBN: Cumple	Cumple
Quasfar	Valoración de CBD Total (UHPLC)	Contenido Total CBD: (CBD + CBDA x 0.877) 10.0% P/V<br>No menos del 90% y no más del 110% del valor declarado	M1 CBD (Conc): 9.51 % p/v<br>M1 CBD (%): 95.10 %<br>M2 CBD (Conc): 9.56 % p/v<br>M2 CBD (%): 95.60 %<br>Promedio CBD: 9.53 % p/v<br>Promedio CBD (%): 95.30 %<br>RSD CBD (%): 0.4 %<br>Promedio CBDA: 0.00 % p/v<br>Promedio CBDA (%): 0.00 %<br>Total CBD (Conc): 9.53 % p/v<br>Total CBD (%): 95.30 %	Cumple
Quasfar	Valoración de THC Total (UHPLC)	Contenido total de THC (THC + THCA x 0.877) < 1 % P/V	Promedio THCd9: 0.12 % p/v<br>Promedio THCA: 0.00 % p/v<br>Total THC (Conc): 0.12 % p/v	Cumple
Quasfar	Valoración de CBN (UHPLC)	NMT 1.0% de CBN	Promedio CBN: 0.00 % p/v	Cumple
USP Vigente	Mesófilos aerobios	<10^3 UFC/g	Resultado: <10 UFC/g	Cumple

Av. Cra. 68 No. 17-90 Piso 2
PBX: 447 0047
Bogotá, D.C. - Colombia
www.quasfar.com.co

MÉTODO	ENSAYO	ESPECIFICACIÓN	RESULTADO	CONCEPTO
USP Vigente	Hongos y Levaduras	<10^2 UFC/g	Resultado: <10 UFC/g	Cumple
USP Vigente	E.coli	Ausencia	Conclusión: Ausente	Cumple
USP Vigente	Salmonella sp	Ausencia	Conclusión: Ausente	Cumple
USP Vigente	Bacterias gram negativas tolerantes a bilis	<10^2 UFC/g	Resultado: <10 UFC/g	Cumple

Concepto Final: LA MUESTRA ANALIZADA CUMPLE CON LAS ESPECIFICACIONES DEL LABORATORIO PROPIETARIO PARA LOS ENSAYOS REALIZADOS.

Anexos:

Se adjuntan las hojas de ruta (hojas de datos primarios de los análisis) las cuales hacen parte integral del presente informe en las que se puede verificar la trazabilidad de los estándares, reactivos, medios de cultivo, materiales de vidrio, equipos usados en cada uno de los análisis, así como las fechas de inicio de incubación y lecturas de los an álisis microbiológicos; en caso que aplique (n).

Analista(s) Responsable(s) (código): 74, 117

Revisó / Aprobó:

Mercedes Ferrer

Director Técnico y de Estudios

Nuestros procesos analíticos están respaldados por Labware LIMS, sistema de información que sigue estricto cumplimiento, trazabilidad, integridad y seguridad de la información, cumpliendo con FDA CFR 21 parte 11.

Este certificado no puede ser reproducido sin previa autorización escrita de la Dirección Técnica de Quasfar M&F S.A.
Los resultados son válidos para la muestra analizada y no para otra de la misma procedencia.

Av. Cra. 68 No. 17-90 Piso 2
PBX: 447 0047
Bogotá, D.C. - Colombia
www.quasfar.com.co

HOJA DE CALCULO QUASFAR M&F S.A.
CONTENIDO DE FITOCANNABINOIDES

Código: VD-005

Revisión: 01

Nombre Del Producto: CANNABIS PREPARACIÓN MAGISTRAL CBD 10%
Tipo De Muestra: Preparación Magistral
Lote: 15-FT-A-0001230620-1-3
Muestra: 128328

ESTANDAR - CURVA DE CALIBRACION

FITOCANNABIDOIDES	Ácido Cannabidiólico (CBDA)		Cannabinol (CBN)		9-Tetrahidrocannabinol (Δ9-THC)		Ácido Tetrahidrocannabinolico A (THCA-A)		Cannabidiol (CBD)	
Alicuota del Estandar / 0,3mL	Concentracion µg/mL	Area (Astd)	Concentracion µg/mL	Area (Astd)	Concentracion µg/mL	Area (Astd)	Concentracion µg/mL	Area (Astd)	Concentracion µg/mL	Area (Astd)
0,010	8,33	17671	8,33	17717	8,33	7724	8,33	17386	8,33	11186
0,010	8,33	17803	8,33	17837	8,33	7419	8,33	17868	8,33	11688
0,010	8,33	17800	8,33	17345	8,33	7633	8,33	18199	8,33	11524
0,030	25,00	54490	25,00	105908	25,00	27088	25,00	47589	25,00	43954
0,030	25,00	53594	25,00	107278	25,00	26698	25,00	45917	25,00	43534
0,030	25,00	53034	25,00	103783	25,00	27630	25,00	47670	25,00	43239
0,060	50,00	108755	50,00	144817	50,00	56849	50,00	98810	50,00	64624
0,060	50,00	109672	50,00	145132	50,00	56857	50,00	99283	50,00	64837
0,060	50,00	109375	50,00	146091	50,00	57935	50,00	99045	50,00	64809
0,140	116,67	256221	116,67	310232	116,67	120292	116,67	231619	116,67	135900
0,140	116,67	253892	116,67	312685	116,67	121098	116,67	227881	116,67	135582
0,140	116,67	254431	116,67	312912	116,67	120529	116,67	232136	116,67	134768
0,280	233,33	526378	233,33	670549	233,33	261264	233,33	481054	233,33	298069
0,280	233,33	529153	233,33	670792	233,33	266706	233,33	476780	233,33	297762
0,280	233,33	527501	233,33	663857	233,33	266706	233,33	479230	233,33	295761
**Intersecciòn (b)**	**-3676,4886**		**7592,8989**		**-2513,2454**		**-3405,7595**		**3245,7903**	
**Pendiente (m)**	**2265,3210**		**2794,6473**		**1130,8667**		**2055,0411**		**1237,3463**	
**Coef. Correlaciòn (r)**	**0,9999**		**0,9971**		**0,9987**		**0,9998**		**0,9974**	
**Coef. Determinaciòn (r2)**	**0,9997**		**0,9942**		**0,9975**		**0,9995**		**0,9948**	

MUESTRA 1 - CONCENTRADA

Activo	Area (Am)		Promedio	RSD ≤ 10%	Concentracion mg/mL	%
Lectura	1	2				
Cannabinol (CBN)	10158	9970	10064	1,3	I.L.C	I.L.C
THCA-A	No detectado	No detectado	N.A	N.A	N.A	No detectado
Ácido Cannabidiólico (CBDA)	8726	8928	8827	1,6	I.L.C	I.L.C
Δ9-THC	12211	12071	12141	0,8	1,17	0,12

MUESTRA 1

Activo	Area (Am)		Promedio	RSD ≤ 10%	Concentracion mg/mL	%	% (Cant Etiq)
Lectura	1	2					
Cannabidiol (CBD)	68870	68377	68624	0,5	95,09	9,51	**95,1**

MUESTRA 2

Activo	Area (Am)		Promedio	RSD ≤ 10%	Concentracion mg/mL	%	% (Cant Etiq)
Lectura	1	2					
Cannabidiol (CBD)	69956	71087	70522	1,1	95,57	9,56	**95,6**

I.L.C. Inferior al límite de cuantificación

MUESTRA	Concentracion mg/mL	%
**mg/mL Cannabidiol (CBD) Promedio (M1-M2-M3)**	95,33	**9,53**
**% Cannabidiol (CBD) (Respecto a lo etiquetado)**	**95,3**	
**RSD**	**0,36**	
**Cannabinol (CBN)**	**I.L.C**	**I.L.C**
**THCA-A**	No detectado	No detectado
**Ácido Cannabidiólico (CBDA)**	**I.L.C**	**I.L.C**
**Δ9-THC**	**1,17**	**0,12**

DATOS

**Fitocannabinoides - Potencia estándar / Concentracion estándar**	**(µg/mL)**
**Estándar Ácido Cannabidiólico (CBDA) Lote:**	**0557556**
**Estándar Cannabinol (CBN) Lote:**	**0546195**
**Estándar 9-Tetrahidrocannabinol (Δ9-THC) Lote:**	**0581756**
**Estándar Ácido Tetrahidrocannabinolico A (THCA-A) Lote:**	**0558070**
**Estándar Cannabidiol (CBD) Lote:**	**0586575**
Ácido Cannabidiólico (CBDA)	1000,0
Cannabinol (CBN)	1000,0
Δ9-Tetrahidrocannabinol (Δ9-THC)	1000,0
Ácido A-Tetrahidrocannabinólico (THCA-A)	1000,0
Cannabidiol (CBD)	1000,0
Factor de Dilución Muestra - Concentrada (10 mL) (Dm)	10,0
Factor de Dilución Muestra - Diluida ((10x10)/0.5) (Dm)	200,0
Peso de Muestra 1 (Pm) mg	103,49
Peso de Muestra 2 (Pm) mg	105,95
Dfensidad (d) (g/mL)	0,9312
Cantidad Etiquetada (g/100mL) (Cant Etiq) (%)	10,0

**CALCULOS**
Concentración (mg fitocannabidoides/mL) = ((Am - b)*Dm*d) / (m*Pm)
% = (Concentración (mg/mL) x 100 g) / 1000 mg
% (Respecto a lo etiquetado) = (%*100)/Cant Etiq

Analista

Edinson Murcia Riaño

2020-07-13
