# Supplementary material for: Sex-Dependent Prescription Patterns and Clinical Outcomes Associated With the Use of Two Oral Cannabis Formulations in the Multimodal Management of Chronic Pain Patients in Colombia
Source: Front Pain Res (Lausanne). 2022 Mar 24;3:854795. doi: 10.3389/fpain.2022.854795 (PMC8987276; doi:10.3389/fpain.2022.854795)
Supplement: Supplementary file 5 [file Data_Sheet_5.PDF]

Sample Information

Sample Name	: A120-1
Sample ID	:
Data File	: A120-1.lcd
Method File	: Cannabinoid_Method_lowTHC.lcm
Date Acquired	: 1/4/2022 6:08:13 PM
Date Processed	: 1/5/2022 2:45:24 PM

Chromatogram

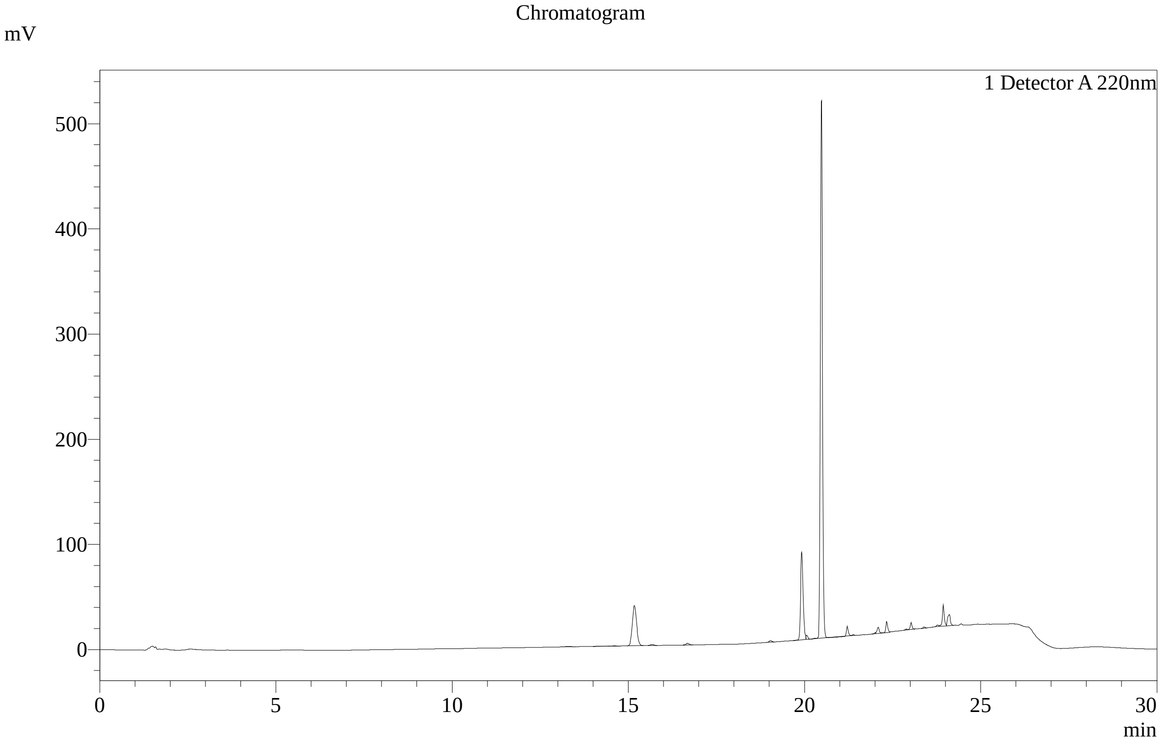

QuantitativeResult

Detector A

ID#	Name	Type	Ret. Time	Conc.	Unit
1	CBDV	Target	14.611	0.022	% w/w
2	PHENANTRENE	I.STD	15.174	0.000	% w/w
3	CBDVA	Target	16.683	0.069	% w/w
4	THCV	Target	--	--	% w/w
5	CBD	Target	19.922	4.697	% w/w
6	CBG	Target	20.057	0.167	% w/w
7	CBDA	Target	20.479	15.108	% w/w
8	CBGA	Target	21.214	0.223	% w/w
9	CBN	Target	--	--	% w/w
10	THC	Target	22.332	0.500	% w/w
11	THCVA	Target	--	--	% w/w
12	CBC	Target	23.026	0.301	% w/w
13	CBNA	Target	23.393	0.039	% w/w
14	THCA	Target	23.938	0.542	% w/w
15	CBCA	Target	24.108	1.163	% w/w
Total				22.831	
