# Supplementary material for: Sex-Dependent Prescription Patterns and Clinical Outcomes Associated With the Use of Two Oral Cannabis Formulations in the Multimodal Management of Chronic Pain Patients in Colombia
Source: Front Pain Res (Lausanne). 2022 Mar 24;3:854795. doi: 10.3389/fpain.2022.854795 (PMC8987276; doi:10.3389/fpain.2022.854795)
Supplement: Supplementary file 6 [file Data_Sheet_6.PDF]

Sample Information

Sample Name	: A120 real
Sample ID	:
Method File	: CANNABIS TERPENES.gcm
Date Acquired	: 1/4/2022 6:58:06 AM
Date Processed	: 1/4/2022 12:44:54 PM

Chromatogram

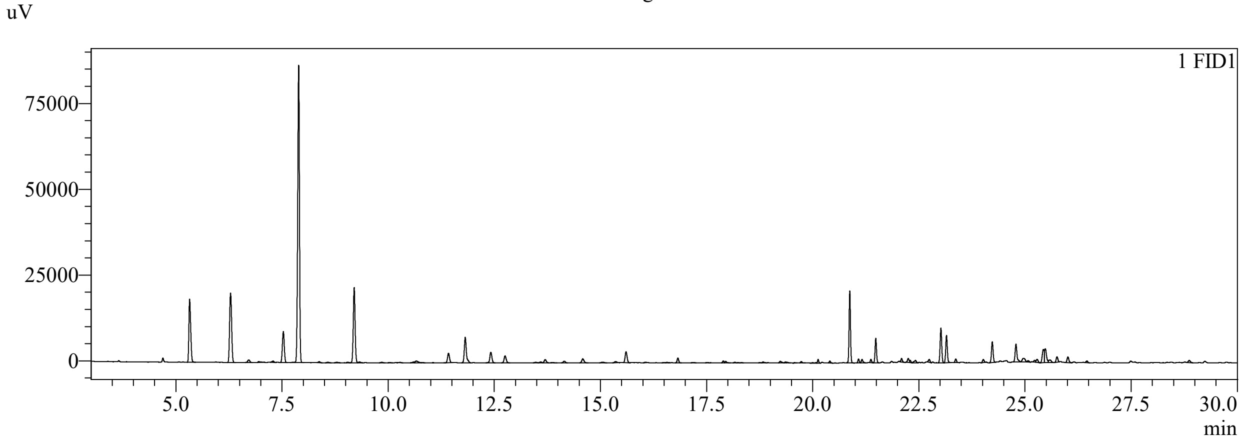

QuantitativeResult

FID1

Name	Ret. Time	Conc.	Unit	Type
Nonane	5.324	0.000	% w/w	I.STD
a-pinene	6.288	0.158	% w/w	Target
Camphene	6.717	0.006	% w/w	Target
Sabinene	7.288	0.003	% w/w	Target
b-pinene	7.531	0.066	% w/w	Target
b-myrcene	7.892	0.578	% w/w	Target
a-phellandrene	8.375	0.002	% w/w	Target
d-3-carene	8.578	0.001	% w/w	Target
a-terpinene	8.790	0.001	% w/w	Target
Limonene	9.199	0.153	% w/w	Target
Eucalyptol	9.326	0.002	% w/w	Target
b-ocimene	9.850	0.001	% w/w	Target
g-terpinene	10.282	0.001	% w/w	Target
Terpinolene	11.421	0.021	% w/w	Target
Linalool	11.815	0.058	% w/w	Target
Fenchol	12.419	0.025	% w/w	Target
Isopulegol	13.698	0.008	% w/w	Target
Borneol	14.584	0.010	% w/w	Target
Menthol	15.044	0.002	% w/w	Target
a-terpineol	15.602	0.024	% w/w	Target
Nerol	16.824	0.008	% w/w	Target
Citronellol	--	--	% w/w	Target
Pulegone	17.521	0.001	% w/w	Target
Geraniol	17.894	0.003	% w/w	Target
Trans-anethole	18.833	0.002	% w/w	Target
Geranyl acetate	19.730	0.002	% w/w	Target
b-elemene	20.404	0.003	% w/w	Target
a-cedrene	--	--	% w/w	Target
b-caryophyllene	20.873	0.111	% w/w	Reference
g-elemene	21.079	0.006	% w/w	Target
a-bergamotene	21.162	0.006	% w/w	Target
a-humulene	21.485	0.039	% w/w	Target
a-amorphene	22.093	0.005	% w/w	Target
a-selinene	22.251	0.007	% w/w	Target
b-selinene	22.303	0.003	% w/w	Target
a-farnesene	22.421	0.006	% w/w	Target
Isocaryophyllene	--	--	% w/w	Target
g-maaliene	22.744	0.005	% w/w	Target
b-maaliene	--	--	% w/w	Target
Aromadendrene	23.020	0.063	% w/w	Target
Eudesma-3,7(11)-diene	23.152	0.049	% w/w	Target
Trans-nerolidol	23.369	0.006	% w/w	Target
Caryophyllene oxide	24.018	0.005	% w/w	Target
Guaiol	24.230	0.038	% w/w	Target
g-eudesmol	24.788	0.036	% w/w	Target
a-eudesmol	25.434	0.029	% w/w	Target
b-eudesmol	25.478	0.030	% w/w	Target
Bulnesol	25.602	0.007	% w/w	Target
a-bisabolol	25.754	0.010	% w/w	Target
Eudesm-7(11)-en-4-ol	26.011	0.012	% w/w	Target
Farnesol	26.458	0.003	% w/w	Target
		1.617		
