# Supplementary material for: Sex-Dependent Prescription Patterns and Clinical Outcomes Associated With the Use of Two Oral Cannabis Formulations in the Multimodal Management of Chronic Pain Patients in Colombia
Source: Front Pain Res (Lausanne). 2022 Mar 24;3:854795. doi: 10.3389/fpain.2022.854795 (PMC8987276; doi:10.3389/fpain.2022.854795)
Supplement: Supplementary file 7 [file Data_Sheet_7.PDF]

Cannabinoid analysis

Inflorescence sample fractions were dried overnight at 50 ºC to remove humidity and crashed and sieved to eliminate any potential seeds and branches from interfering the results. Around 0.1 g of sieved plant material or oil were weighed in glass vials and after adding 5 mL of EtOH with phenanthrene at 300 ppm, they were sonicated during 15 min. Afterwards, samples were diluted in a 1:30 ratio, filtered and injected.

Cannabinoid analysis was performed by a high-performance liquid chromatography (HPLC) coupled to a UV detector, in a Shimadzu Prominence-i LC-2030 plus system. The analysis was performed according to Aizpurua-Olaizola et al., (2016) method with some modifications. Chromatographic separation was achieved using a Nexleaf CBX (2.7 µm, 150 mm x 4.6 mm i.d.) with a Nexleaf CBX guard column (2.7 µm) and a binary A/B gradient (solvent A was H2O with 0.085 % of ortophosphoric acid, and solvent B was MeOH with 0.085 % of ortophosphoric acid). The gradient program was as follows: start with 60% B and hold it for 5 min, then gradually increase to 72% B in 11 min, then increase it to 95% B in 6 min and hold it for 2 min, finally decrease it back to 60% B in 1 min and hold it for 5 min for equilibration. A flow rate of 1 mL/min was used, the column was set at 50 °C, and the injection volume was 5 µL. Cannabinoids were quantified at a detection wavelength of 220 nm.

Quantification was performed using an external calibration, by the average values of two sets of analytical standards with concentrations ranging from 0.1 to 200 ppm. Low and high calibration ranges were used in each set, i.e., 0.1-10 ppm and 5-200 µg/mL, respectively. System fluctuations were corrected with the internal standard phenanthrene, and quality control samples were injected along with the samples.

Terpene analysis

Around 0.4g of air-dried plant or oil were weighed in glass vials and 4.5 mL internal standard solution of nonane were added, ultrasonicated for 30 minutes and centrifugated for 5 minutes. Terpenes' analysis was performed via gas chromatography-flame ionization detector (GC-FID) using a Shimadzu GC-2010 pro instrument equipped with an SH-Rxi-5ms column (0.25 µm, 30 m x 0.25 mm i.d.) from Shimadzu.

The analysis was performed according to Aizpurua-Olaizola et al., (2016) with minor modifications. The injector temperature was set to 250 °C, the injection volume was 1 µL, and a split ratio of 1:10 was used. A carrier gas (H2) flow rate of 1.12 mL/min was used. The oven temperature program started at 60 °C with a ramp rate of 3 °C/min until 106 °C was reached, then the temperature was increased to 160 °C with a ramp rate of 12, following with a increase to 202 °C with a ramp rate of 3 °C/min; then the temperature was increased to 320 °C with a ramp rate of 40 °C/min and hold it for 3 min for cleaning purposes. The FID detector temperature was set to 330 °C. A H2 flow of 40 mL/min and synthetic air flow of 400 mL/min were used.

Because of the low variability in the response factors of compounds with similar molecular mass (Aizpurua-Olaizola et al., 2016), terpene quantification was performed using the average of three calibration sets containing γ-terpinene at concentrations ranging from 1 to 2000 µg/mL in EtOH. System fluctuations were corrected with the

internal standard, nonane, at 100 µg/mL, and quality control samples were injected with the samples.

A GC coupled to a mass spectrometer (GC-MS) was used for compound identification. Similar oven gradient and injection and flow conditions used GC-FID were applied, but in this case, helium was used as the carrier gas instead of hydrogen. The NIST library (Standard References Data Program of the National Institute of Standards and Technology, distributed by Agilent Technologies) was used for compound identification. The terpenes α-pinene, camphene, sabinene, β-pinene, β-myrcene, α-phellandrene, 3-carene, α-terpinene, limonene, eucalyptol, β-ocimene, γ-terpinene, terpinolene, linalool, fenchol, isopulegol, borneol, menthol, γ-terpineol, nerol, citronellol, pulegone, geraniol, trans anethole, geranyl Acetate, β-elemene, α-cedrene, β-caryophyllene, α-humulene, trans nerolidol, caryophyllene oxide, guaiol, β-eudesmol, α-bisabolol were verified by comparing their retention times and the obtained mass spectra with reference standards, whereas γ-elemene, α-bergamotene, α-amorphene, α-selinene, β-selinene, α-farnesene, isocaryophillene, γ-maaliene, β-maaliene, aromadendrene, eudesma-3,7(11)-diene, γ-eudesmol, α-eudesmol, bulnesol, eudesm-7(11)-en-4-ol and farnesol were identified using the NIST library.

O. Aizpurua-Olaizola et al. Evolution of the Cannabinoid and Terpene Content during the Growth of Cannabis sativa Plants from Different Chemotypes. J Nat Prod (2016), 79, 324-31. doi: 10.1021/acs.jnatprod.5b00949
