# Supplementary material for: Sex-Dependent Prescription Patterns and Clinical Outcomes Associated With the Use of Two Oral Cannabis Formulations in the Multimodal Management of Chronic Pain Patients in Colombia
Source: Front Pain Res (Lausanne). 2022 Mar 24;3:854795. doi: 10.3389/fpain.2022.854795 (PMC8987276; doi:10.3389/fpain.2022.854795)
Supplement: Supplementary file 8 [file Data_Sheet_8.PDF]

Sample Information

Sample Name	: A118-1
Sample ID	:
Data File	: A118-1.lcd
Method File	: Cannabinoid_Method_highTHC.lcm
Date Acquired	: 1/4/2022 5:07:15 PM
Date Processed	: 1/5/2022 2:44:51 PM

Chromatogram

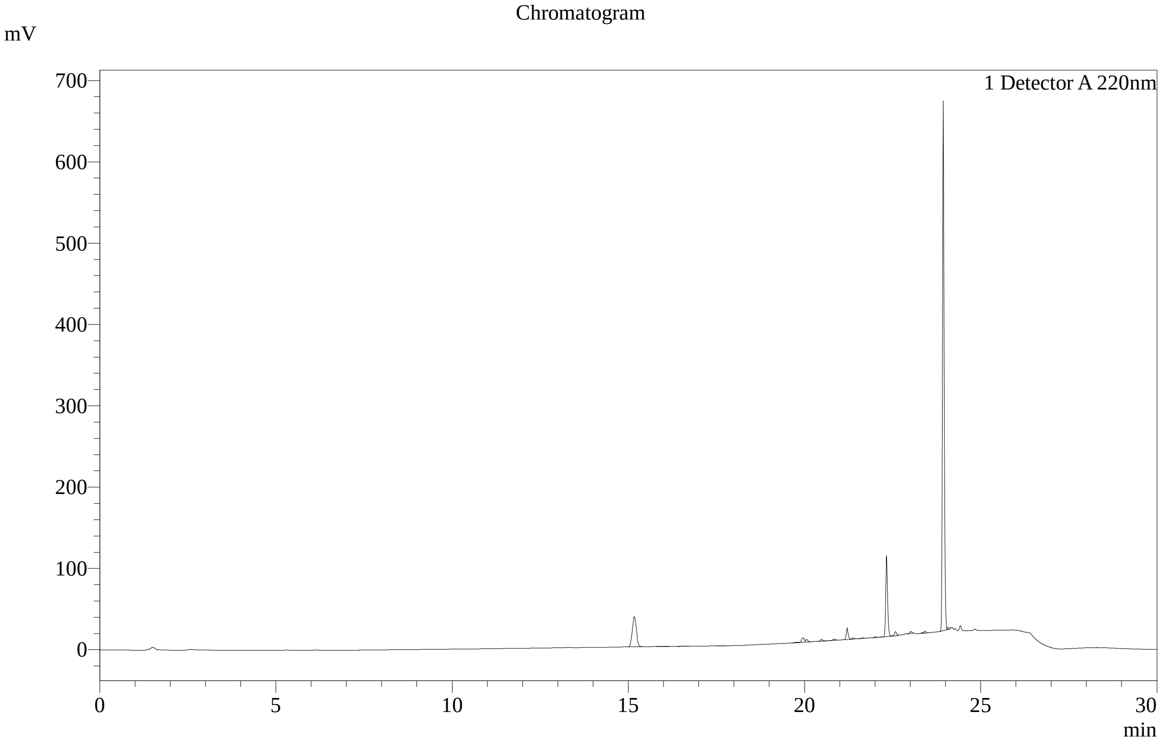

QuantitativeResult

Detector A

ID#	Name	Type	Ret. Time	Conc.	Unit
1	CBDV	Target	--	--	% w/w
2	PHENANTRENE	I.STD	15.171	0.000	% w/w
3	CBDVA	Target	16.516	0.008	% w/w
4	THCV	Target	19.742	0.050	% w/w
5	CBD	Target	19.959	0.361	% w/w
6	CBG	Target	20.063	0.172	% w/w
7	CBDA	Target	20.480	0.075	% w/w
8	CBGA	Target	21.210	0.400	% w/w
9	CBN	Target	21.652	0.021	% w/w
10	THC	Target	22.329	4.469	% w/w
11	THCVA	Target	22.582	0.161	% w/w
12	CBC	Target	23.019	0.129	% w/w
13	CBNA	Target	23.345	0.038	% w/w
14	THCA	Target	23.937	16.406	% w/w
15	CBCA	Target	24.073	0.142	% w/w
Total				22.432	
