# Supplementary material for: Sex-Dependent Prescription Patterns and Clinical Outcomes Associated With the Use of Two Oral Cannabis Formulations in the Multimodal Management of Chronic Pain Patients in Colombia
Source: Front Pain Res (Lausanne). 2022 Mar 24;3:854795. doi: 10.3389/fpain.2022.854795 (PMC8987276; doi:10.3389/fpain.2022.854795)
Supplement: Supplementary file 9 [file Data_Sheet_9.PDF]

Sample Information

Sample Name	: A118 real
Sample ID	:
Method File	: CANNABIS TERPENES.gcm
Date Acquired	: 1/4/2022 6:11:19 AM
Date Processed	: 1/4/2022 12:06:56 PM

Chromatogram

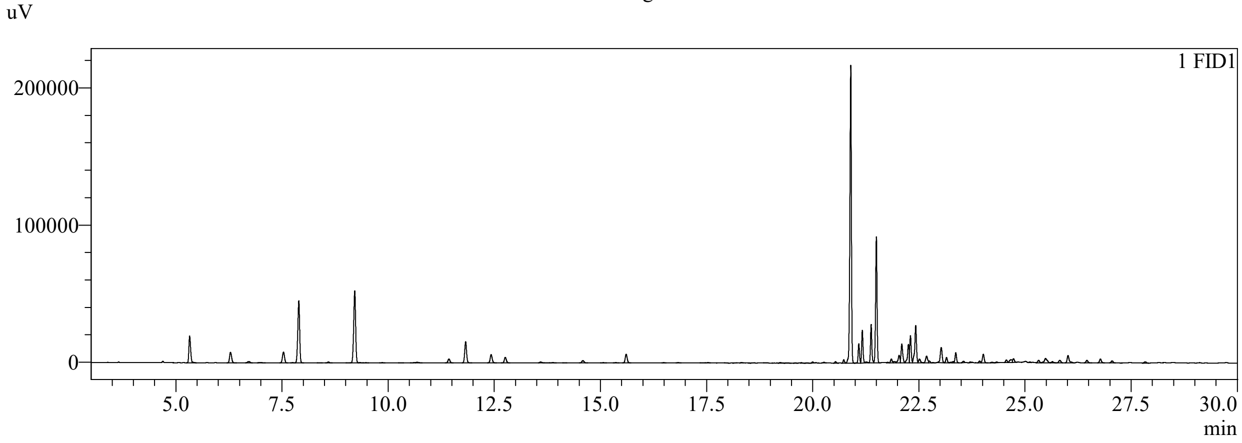

QuantitativeResult

FID1

Name	Ret. Time	Conc.	Unit	Type
Nonane	5.324	0.000	% w/w	I.STD
a-pinene	6.285	0.058	% w/w	Target
Camphene	6.717	0.008	% w/w	Target
Sabinene	--	--	% w/w	Target
b-pinene	7.535	0.061	% w/w	Target
b-myrcene	7.895	0.315	% w/w	Target
a-phellandrene	8.377	0.001	% w/w	Target
d-3-carene	8.592	0.004	% w/w	Target
a-terpinene	--	--	% w/w	Target
Limonene	9.213	0.378	% w/w	Target
Eucalyptol	9.338	0.001	% w/w	Target
b-ocimene	9.860	0.001	% w/w	Target
g-terpinene	10.293	0.001	% w/w	Target
Terpinolene	11.431	0.021	% w/w	Target
Linalool	11.825	0.108	% w/w	Target
Fenchol	12.426	0.044	% w/w	Target
Isopulegol	13.706	0.001	% w/w	Target
Borneol	14.586	0.014	% w/w	Target
Menthol	15.070	0.001	% w/w	Target
a-terpineol	15.606	0.046	% w/w	Target
Nerol	16.833	0.002	% w/w	Target
Citronellol	17.397	0.001	% w/w	Target
Pulegone	17.537	0.002	% w/w	Target
Geraniol	17.955	0.001	% w/w	Target
Trans-anethole	18.876	0.000	% w/w	Target
Geranyl acetate	19.733	0.001	% w/w	Target
b-elemene	--	--	% w/w	Target
a-cedrene	20.731	0.012	% w/w	Target
b-caryophyllene	20.896	1.220	% w/w	Reference
g-elemene	21.086	0.072	% w/w	Target
a-bergamotene	21.168	0.122	% w/w	Target
a-humulene	21.499	0.488	% w/w	Target
a-amorphene	22.099	0.086	% w/w	Target
a-selinene	22.255	0.085	% w/w	Target
b-selinene	22.302	0.103	% w/w	Target
a-farnesene	22.426	0.179	% w/w	Target
Isocaryophyllene	22.515	0.021	% w/w	Target
g-maaliene	22.682	0.038	% w/w	Target
b-maaliene	22.844	0.002	% w/w	Target
Aromadendrene	23.027	0.079	% w/w	Target
Eudesma-3,7(11)-diene	23.152	0.022	% w/w	Target
Trans-nerolidol	23.370	0.042	% w/w	Target
Caryophyllene oxide	24.019	0.042	% w/w	Target
Guaiol	24.211	0.003	% w/w	Target
g-eudesmol	24.730	0.017	% w/w	Target
a-eudesmol	25.485	0.034	% w/w	Target
b-eudesmol	--	--	% w/w	Target
Bulnesol	25.645	0.005	% w/w	Target
a-bisabolol	25.821	0.014	% w/w	Target
Eudesm-7(11)-en-4-ol	26.014	0.042	% w/w	Target
Farnesol	26.459	0.013	% w/w	Target
		3.816		
