# Supplementary material for: Sex-Dependent Prescription Patterns and Clinical Outcomes Associated With the Use of Two Oral Cannabis Formulations in the Multimodal Management of Chronic Pain Patients in Colombia
Source: Front Pain Res (Lausanne). 2022 Mar 24;3:854795. doi: 10.3389/fpain.2022.854795 (PMC8987276; doi:10.3389/fpain.2022.854795)
Supplement: Supplementary file 10 [file Data_Sheet_10.PDF]

Sample Information

Sample Name	: A116-1
Sample ID	:
Data File	: A116-1.lcd
Method File	: Cannabinoid_Method_highTHC.lcm
Date Acquired	: 1/4/2022 4:06:17 PM
Date Processed	: 1/5/2022 2:44:09 PM

Chromatogram

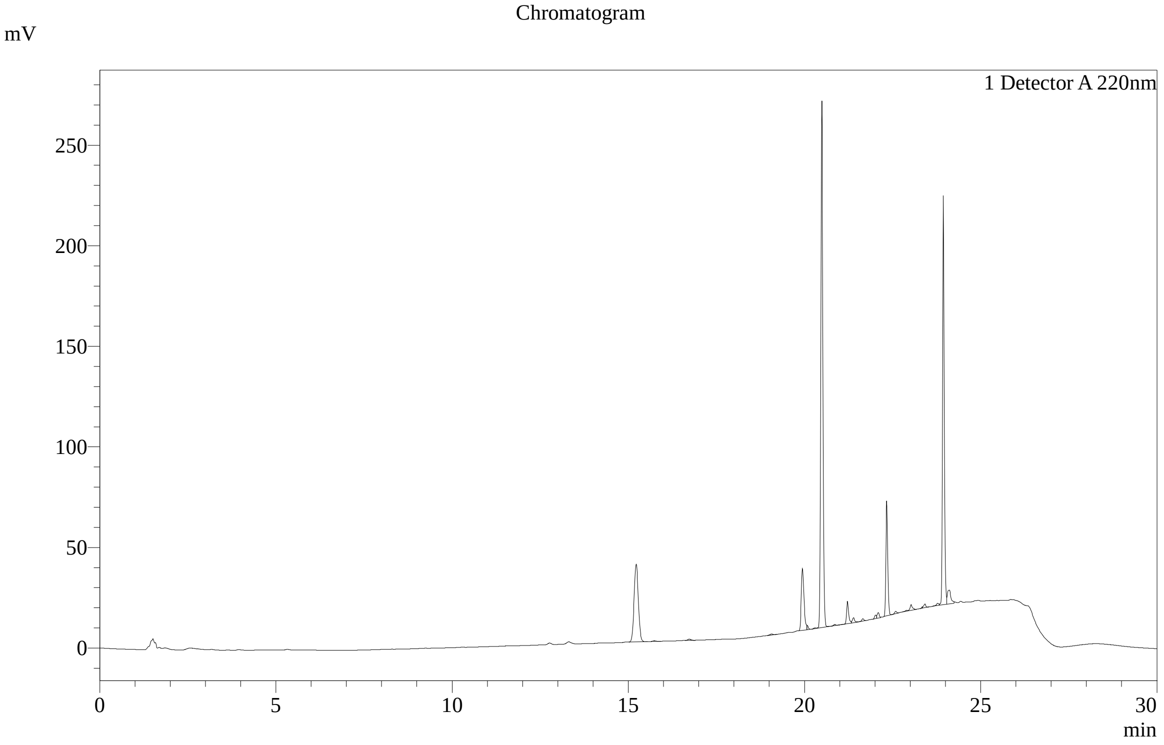

QuantitativeResult

Detector A

ID#	Name	Type	Ret. Time	Conc.	Unit
1	CBDV	Target	--	--	% w/w
2	PHENANTRENE	I.STD	15.224	0.000	% w/w
3	CBDVA	Target	16.732	0.025	% w/w
4	THCV	Target	--	--	% w/w
5	CBD	Target	19.942	1.842	% w/w
6	CBG	Target	20.071	0.070	% w/w
7	CBDA	Target	20.492	7.614	% w/w
8	CBGA	Target	21.220	0.319	% w/w
9	CBN	Target	21.658	0.033	% w/w
10	THC	Target	22.334	2.354	% w/w
11	THCVA	Target	22.588	0.029	% w/w
12	CBC	Target	23.026	0.166	% w/w
13	CBNA	Target	23.346	0.013	% w/w
14	THCA	Target	23.940	4.879	% w/w
15	CBCA	Target	24.109	0.963	% w/w
Total				18.307	
