# Supplementary material for: Sex-Dependent Prescription Patterns and Clinical Outcomes Associated With the Use of Two Oral Cannabis Formulations in the Multimodal Management of Chronic Pain Patients in Colombia
Source: Front Pain Res (Lausanne). 2022 Mar 24;3:854795. doi: 10.3389/fpain.2022.854795 (PMC8987276; doi:10.3389/fpain.2022.854795)
Supplement: Supplementary file 11 [file Data_Sheet_11.PDF]

Sample Information

Sample Name	: A116 real
Sample ID	:
Method File	: CANNABIS TERPENES.gcm
Date Acquired	: 1/4/2022 5:24:28 AM
Date Processed	: 1/4/2022 12:08:17 PM

Chromatogram

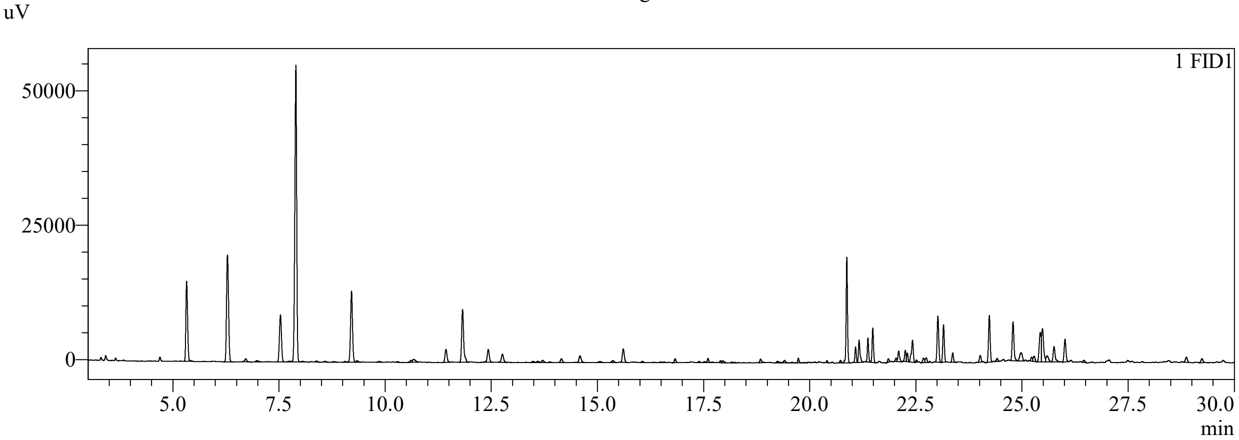

QuantitativeResult

FID1

Name	Ret. Time	Conc.	Unit	Type
Nonane	5.323	0.000	% w/w	I.STD
a-pinene	6.285	0.185	% w/w	Target
Camphene	6.716	0.005	% w/w	Target
Sabinene	--	--	% w/w	Target
b-pinene	7.533	0.082	% w/w	Target
b-myrcene	7.896	0.470	% w/w	Target
a-phellandrene	8.387	0.002	% w/w	Target
d-3-carene	8.587	0.001	% w/w	Target
a-terpinene	8.798	0.001	% w/w	Target
Limonene	9.208	0.119	% w/w	Target
Eucalyptol	9.337	0.002	% w/w	Target
b-ocimene	9.863	0.001	% w/w	Target
g-terpinene	10.294	0.001	% w/w	Target
Terpinolene	11.432	0.022	% w/w	Target
Linalool	11.823	0.091	% w/w	Target
Fenchol	12.428	0.023	% w/w	Target
Isopulegol	13.709	0.004	% w/w	Target
Borneol	14.589	0.012	% w/w	Target
Menthol	15.055	0.003	% w/w	Target
a-terpineol	15.608	0.022	% w/w	Target
Nerol	16.829	0.005	% w/w	Target
Citronellol	17.392	0.001	% w/w	Target
Pulegone	17.522	0.001	% w/w	Target
Geraniol	17.898	0.002	% w/w	Target
Trans-anethole	18.842	0.005	% w/w	Target
Geranyl acetate	19.731	0.006	% w/w	Target
b-elemene	20.406	0.002	% w/w	Target
a-cedrene	20.724	0.003	% w/w	Target
b-caryophyllene	20.874	0.123	% w/w	Reference
g-elemene	21.080	0.018	% w/w	Target
a-bergamotene	21.163	0.029	% w/w	Target
a-humulene	21.485	0.041	% w/w	Target
a-amorphene	22.095	0.015	% w/w	Target
a-selinene	22.251	0.014	% w/w	Target
b-selinene	22.299	0.010	% w/w	Target
a-farnesene	22.421	0.036	% w/w	Target
Isocaryophyllene	22.517	0.003	% w/w	Target
g-maaliene	22.682	0.007	% w/w	Target
b-maaliene	22.744	0.007	% w/w	Target
Aromadendrene	23.019	0.065	% w/w	Target
Eudesma-3,7(11)-diene	23.152	0.049	% w/w	Target
Trans-nerolidol	23.369	0.012	% w/w	Target
Caryophyllene oxide	24.017	0.010	% w/w	Target
Guaiol	24.231	0.065	% w/w	Target
g-eudesmol	24.789	0.059	% w/w	Target
a-eudesmol	25.430	0.051	% w/w	Target
b-eudesmol	25.483	0.058	% w/w	Target
Bulnesol	25.593	0.013	% w/w	Target
a-bisabolol	25.756	0.026	% w/w	Target
Eudesm-7(11)-en-4-ol	26.014	0.035	% w/w	Target
Farnesol	26.458	0.003	% w/w	Target
		1.824		
